# The Exopolysaccharide Matrix Modulates the Interaction between 3D Architecture and Virulence of a Mixed-Species Oral Biofilm

**DOI:** 10.1371/journal.ppat.1002623

**Published:** 2012-04-05

**Authors:** Jin Xiao, Marlise I. Klein, Megan L. Falsetta, Bingwen Lu, Claire M. Delahunty, John R. Yates, Arne Heydorn, Hyun Koo

**Affiliations:** 1 Center for Oral Biology, University of Rochester Medical Center, Rochester, New York, United States of America; 2 State Key Laboratory of Oral Diseases, Sichuan University, Chengdu, China; 3 Department of Chemical Physiology, The Scripps Research Institute, La Jolla, California, United States of America; 4 Department of General Medicine, Glostrup Hospital, Glostrup, Denmark; 5 Department of Microbiology and Immunology, University of Rochester Medical Center, Rochester, New York, United States of America; Carnegie Mellon University, United States of America

## Abstract

Virulent biofilms are responsible for a range of infections, including oral diseases. All biofilms harbor a microbial-derived extracellular-matrix. The exopolysaccharides (EPS) formed on tooth-pellicle and bacterial surfaces provide binding sites for microorganisms; eventually the accumulated EPS enmeshes microbial cells. The metabolic activity of the bacteria within this matrix leads to acidification of the milieu. We explored the mechanisms through which the *Streptococcus mutans*-produced EPS-matrix modulates the three-dimensional (3D) architecture and the population shifts during morphogenesis of biofilms on a saliva-coated-apatitic surface using a mixed-bacterial species system. Concomitantly, we examined whether the matrix influences the development of pH-microenvironments within intact-biofilms using a novel 3D *in situ* pH-mapping technique. Data reveal that the production of the EPS-matrix helps to create spatial heterogeneities by forming an intricate network of exopolysaccharide-enmeshed bacterial-islets (microcolonies) through localized cell-to-matrix interactions. This complex 3D architecture creates compartmentalized acidic and EPS-rich microenvironments throughout the biofilm, which triggers the dominance of pathogenic *S. mutans* within a mixed-species system. The establishment of a 3D-matrix and EPS-enmeshed microcolonies were largely mediated by the *S. mutans gtfB/gtfC* genes, expression of which was enhanced in the presence of *Actinomyces naeslundii* and *Streptococcus oralis*. Acidic pockets were found only in the interiors of bacterial-islets that are protected by EPS, which impedes rapid neutralization by buffer (pH 7.0). As a result, regions of low pH (<5.5) were detected at specific locations along the surface of attachment. Resistance to chlorhexidine was enhanced in cells within EPS-microcolony complexes compared to those outside such structures within the biofilm. Our results illustrate the critical interaction between matrix architecture and pH heterogeneity in the 3D environment. The formation of structured acidic-microenvironments in close proximity to the apatite-surface is an essential factor associated with virulence in cariogenic-biofilms. These observations may have relevance beyond the mouth, as matrix is inherent to all biofilms.

## Author Summary

Virulent biofilms formed on surfaces are associated with many human infections. The disease dental caries, expressed as cavities, is a prime example of the consequences arising from interactions between bacteria and sugars on tooth-surfaces. When *Streptococcus mutans* metabolize sugars, they produce a glue-like polymer termed glucan, helping them to adhere firmly to teeth. Glucan is also formed on bacterial surfaces in the mouth, and will accumulate and enmesh additional microorganisms creating the gelatinous formation known as dental plaque-biofilm. We found unique islets of bacteria within these biofilms, particularly close to the tooth-surface, providing safe havens in which bacteria thrive and produce acids that erode teeth. One intriguing mystery is why acids accumulate on the tooth-surface when there is an abundance of neutral-pH saliva surrounding the teeth. We found that bacterial-islets are particularly protected by glucan, which retards neutralization. We noticed that, within biofilms, the interiors of these islets are acidic, where only acid-tolerant bacteria can prosper, ensuring continued localized acid production. Our study demonstrates that construction of biofilms mediated by glucans forms complex 3D architectures, creating a variety of acidic-microenvironments that are essential for virulence expression. These results may aid in the development of enhanced methods to modulate biofilm formation.

## Introduction

Virulent biofilms formed on surfaces are prime biological phenomena that are associated with many illnesses and infections in humans, including oral diseases, native valve endocarditis, and a number of nosocomial infections [1]. Human dental plaque is one of the most complex biofilm systems in nature, as it is constantly exposed to environmental challenges and therefore it may serve as a general model to study the biology of biofilms [2]. Dental caries is a highly prevalent and costly biofilm-dependent oral infectious disease, which continues to be a significant cause of emergency room visits and absences from work or school [3]. This ubiquitous disease results from the complex interactions that occur on tooth surfaces between specific oral bacteria, the products produced by these organisms, salivary constituents, and dietary carbohydrates [4]. These interactions modulate the transition from a healthy condition to a disease state (dental caries), which is characterized by the establishment of cariogenic biofilms on the susceptible tooth surface, and eventually leads to cavitation through acid dissolution of enamel [4].

All bacterial biofilms have at least one property in common: the presence of a bacterial-derived matrix that envelops the cells and helps to facilitate the formation of multicellular structures that are firmly attached to a surface [5]. The composition and structure of the biofilm matrix may be as variable as the microbial constituents, are influenced by local environmental conditions, and can change with time [5], [6]. Exopolysaccharides (EPS), proteins, lipids, nucleic acids, lipoteichoic acids, and even lipopolysaccharides have been identified in the matrices of bacterial biofilms [5], [7]–[9]. The matrix is considered essential for the existence of the biofilm lifestyle and full expression of virulence by bacterial pathogens [5]. However, most previous research has been focused on the (bacterial) cellular aspects of biofilm, while the functional roles that matrices play in the expression of virulence have received limited attention.

Exopolysaccharides are key components of the matrix in cariogenic oral biofilms, and are recognized virulence factors involved in the pathogenesis of dental caries [4]. In the mouth, a highly diverse microbial community is constantly interacting with a proteinaceous film present on the tooth surface, known as the pellicle. The pellicle, to which a small group of organisms (mostly Streptococci and *Actinomyces spp.*) can adhere in low numbers [10]–[12], is derived from mammalian and bacterial sources (such as salivary proteins and exoenzymes). Cariogenic organisms such as *Streptococcus mutans* can also be present in this initial colonizing community, albeit in varying numbers, which is dependent on the host diet [13], [14]. However, environmental changes, such as frequent consumption of sucrose, dramatically influence biofilm development by providing a substrate for EPS production on the pellicle surface [4], [11], [15]. Glucosyltransferases (Gtfs) from *S. mutans* are constituents of the pellicle and are capable of synthesizing glucans *in situ* from sucrose [16], [17]. The surface-formed polymers provide bacterial binding sites for subsequent colonization and local accumulation of *S. mutans* (through several membrane-associated glucan binding proteins) and other organisms [18], [19]. Gtfs also bind many oral bacteria, even those that do not synthesize Gtfs [17], [20], [21], thereby converting them into *de facto* glucan producers [17]. Thus, a bacterial product in the pellicle and on microbial surfaces provides the early foundation of the EPS-rich matrix (and binding sites) [4], [22], thereby dramatically influencing the trajectory of virulent biofilm formation.

In parallel, sucrose can also be fermented to produce acids within biofilms. Other fermentable carbohydrates (e.g. glucose, fructose, and maltose) can also be converted into acids, but rarely serve as a substrate for EPS synthesis [4], [23]. The acidification of the EPS-rich matrix favors the growth of the microorganisms that are tolerant to acidic stress and promotes demineralization of the adjacent tooth enamel, leading to the clinical onset of cavitation [4], [24], [25]. Cariogenic biofilms are therefore comprised of mixed microbiota *in vivo* bound by a common matrix; all of the microorganisms are usually potent producers of acid and are acid-tolerant, which creates a highly adhesive, cohesive, and acidic milieu. How the matrix mediates the construction of the 3D biofilm architecture and establishes spatial heterogeneities, and why acids accumulate within oral biofilms, remain intriguing mysteries, particularly when there is an abundance of buffering saliva that surrounds the teeth and would theoretically be capable of neutralizing these acids.

Matrix constituents, such as EPS, could affect the diffusion of substances in and out of the biofilm, perhaps helping to create a diverse range of microenvironments within the biofilm [26]. However, the knowledge of how polysaccharides are assembled three-dimensionally is limited, particularly due to difficulties in studying the matrix of intact biofilms. The polysaccharides undergo structural modifications resulting from the effects of glucanohydrolases, and may not be evenly or homogeneously distributed within intact plaque-biofilms [4], [27]. Prior investigations that have attempted to characterize the unique microenvironments within biofilms have relied heavily on the use of invasive techniques (e.g. using probes or microsensors) [28], [29], which alter the complex structure and physical properties of the matrix, complicating the interpretation of data. Thus, the precise role of EPS in the formation and structure of acidic environments within biofilms remains largely unexplored [4], [5].

In the present study, we focused on understanding the function of the matrix associated with (1) the construction of the 3D biofilm architecture, and (2) the modulation of the microenvironmental pH in the resultant milieu within a mixed-species system. To achieve these goals, we devised novel approaches by incorporating a fluorophore or fluorescent pH indicator during synthesis of the matrix. This technique allowed us to follow the development of the intricate matrix structure in a sequential fashion and simultaneously map the spatial distribution of pH within the undisturbed 3D biofilm architecture.

## Results/Discussion

Our results illustrate the interaction between the architecture of the biofilm matrix and the heterogeneity of the microenvironment present within mixed-species biofilms. The EPS-rich matrix facilitates the formation of punctate areas of low pH values within intact biofilm, many that are located in close proximity to the apatite-surface. The resultant milieu favors the selection of acidogenic, aciduric, and glucan-binding pathogens (e.g. *S. mutans*), and creates demineralizing sites at the surface of biofilm attachment, thereby shifting from a non-virulent to a virulent phase.

### The assembly of the EPS matrix in 3D

**Figure 1:**
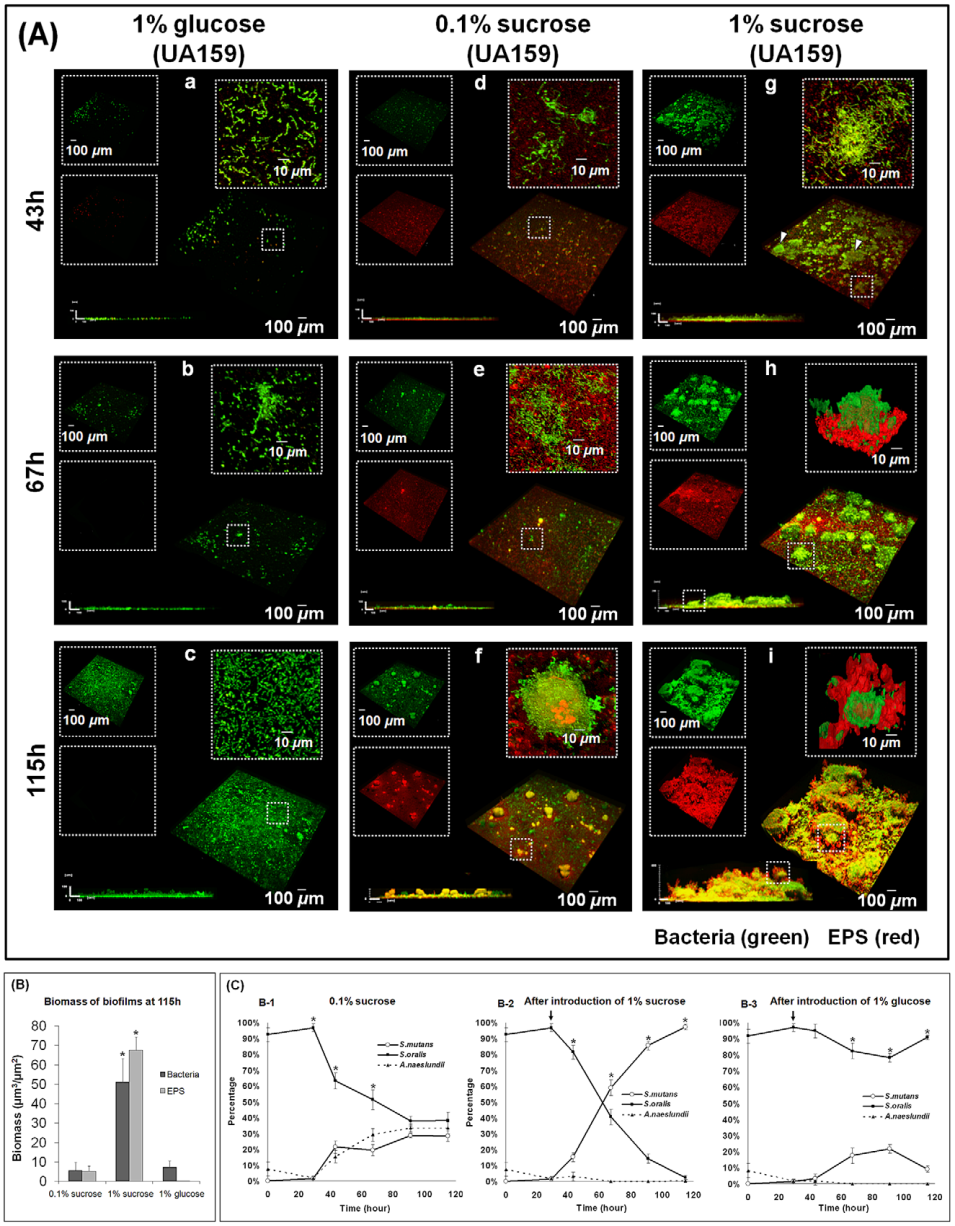
Dynamics of the morphogenesis, 3D architecture development and microbial population shifts of mixed-species biofilms. (**A**) Representative 3D rendering images of mixed-species biofilms at distinct time points. Images (a–c) represent biofilms formed after the introduction of 1% (w/v) glucose. Images (d–f) represent biofilms formed in the presence of 0.1% (w/v) sucrose. Images (g–i) represent biofilms formed after the introduction of 1% (w/v) sucrose. The EPS channel is depicted in red, while the cells are depicted in green. At the upper left of each panel, the two channels are displayed separately, while the merged image is displayed at the bottom right. Lateral (side) views of each biofilm are displayed at the bottom left, while a magnified (close-up) view of each small box depicted in the merged image is positioned in the upper right corner of each panel. (**B**) The data are mean values ± s.d. (n = 30). The asterisks (*) indicate that the each of the values (EPS and bacterial biomass) in the 1% sucrose group are significantly different from others (*P*<0.05, Tukey-Krammer HSD). (**C**) Bacterial species [*S. mutans* (10^2^ CFU/ml), *A. naeslundii* (10^6^ CFU/ml), and *S. oralis* (10^7^ CFU/ml)] were inoculated in the culture medium supplemented with 0.1% (w/v) sucrose until 29 h for establishment of the initial biofilm community [*S. oralis* (5.6±3.5×10^6^ CFU), *A. naeslundii* (9.4±6.5×10^4^ CFU) and *S. mutans* (8.8±2.5×10^3^ CFU)]. The biofilms were then challenged with an environmental change by introducing 1% (w/v) sucrose (B-2) or 1% (w/v) glucose (B-3). Viable populations of *S. mutans*, *S. oralis*, and *A. naeslundii* recovered from the biofilms were counted (number of CFU recovered per biofilm) over time, and the proportion of each strain at each time point was calculated based on CFU data. Microbial population at 115 h in: 1% sucrose (1.1±0.2×10^11^ CFU of *S. mutans*; 2.1±2.9×10^9^ CFU of *S. oralis*; 1.7±0.4×10^7^ CFU of *A. naeslundii*), 0.1% sucrose (6.7±0.8×10^9^ CFU of *S. mutans*; 1.4±1.9×10^10^ CFU of *S. oralis*; 9.3±1.3×10^9^ CFU of *A. naeslundii*), and 1% glucose (2.6±1.9×10^9^ CFU of *S. mutans*; 3.2±0.4×10^10^ CFU of *S. oralis*). The data are mean values ± s.d. (n = 12). The asterisks (*) indicate that the values from each strain are significantly different from each other (*P*<0.05).

We examined over time the morphogenesis of mixed-species biofilms on saliva-coated apatitic (sHA) discs. We focused on the development of the architecture in three-dimensions (3D), which is associated with the early accumulation of EPS on the sHA surface and the subsequent construction of an EPS-rich matrix. The formation and the sequence of assembly of the EPS matrix are heavily influenced by the type of sugars included in the test system (Figure 1A). Following the introduction of 1% sucrose, a heterogeneous layer of EPS is formed on the sHA surface, and localized accumulations of microorganisms are observed on the EPS surface after 43 h (Figure 1Ag; see arrows). After 67 h of development (Figure 1Ah), the cells are densely packed with EPS, forming a 3D bacterial structure (termed EPS-microcolony complex; highlighted in Figure 1Ah). After 115 h, the size of these bacterial structures had increased in several dimensions; they became more enmeshed in, and surrounded by, the EPS-matrix as time elapsed (Figure 1Ai). Two distinct locations of EPS-microcolony complexes were observed: those attached to the EPS layer formed on the sHA (Figure 1Ah), and those tethered to the body of the matrix, near the fluid interface (highlighted in Figure 1Ai). These observations, made when sucrose is abundant (1%) in the test system, are in marked contrast from those made when sucrose was limited (0.1%) or was replaced completely by glucose. A thin EPS layer on the sHA surface, and a few small EPS-attached microcolonies (at later stages) were detected in biofilms formed with 0.1% sucrose (Figure 1Ad–f). When 1% glucose was present, neither an EPS layer nor 3D bacterial islets were assembled. Rather, a thin and mostly homogenous accumulation of bacterial cells adherent to the sHA surface was observed (Figure 1Aa–c). The close-up images reveal that the structural organization of the accumulated cells lacking an EPS matrix is based primarily on cell to cell binding (co-aggregation).

The presence of 1% sucrose also increased the biomass of the biofilms as a consequence of the assembly of an EPS-rich matrix and the development of the architecture in 3D. Sucrose-grown biofilms resulted in thicker biofilms containing up to 10-fold more EPS (and also more bacterial biomass) than those formed with 1% glucose at 115 h (Figure 1B; *P*<0.05). The essential role *S. mutans* and/or its products played in the development of the 3D architecture was revealed by the failure to assemble any matrix or EPS-enmeshed microcolonies when *S. oralis* and *A. naeslundii* were cultured together in the absence of *S. mutans* [22].

**Figure 2:**
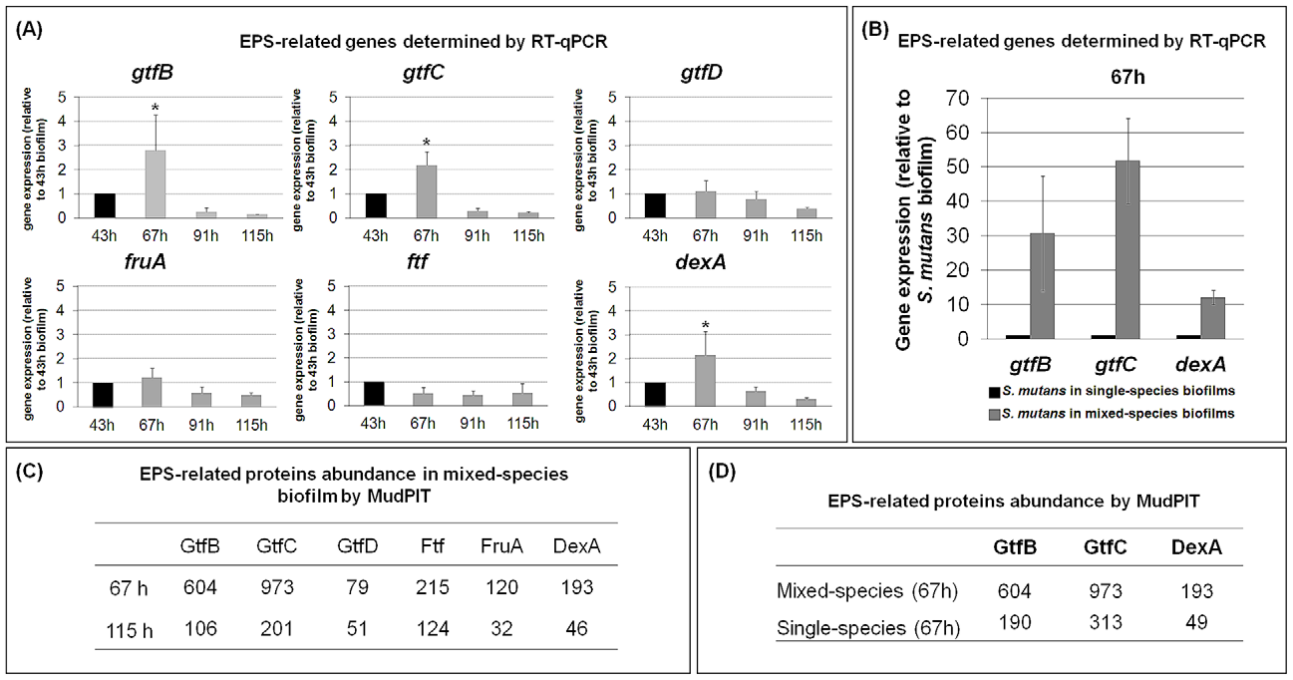
Expression of *Streptococcus mutans* EPS-associated genes and proteins during mixed-species biofilm development. (**A**) This panel shows RT-qPCR analysis of *gtfB*, *gtfC*, *gtfD*, *fruA*, *ftf* and *dexA* gene expression by *S. mutans* in mixed-species biofilms at specific time points after introduction of 1% (w/v) sucrose. The data shown are mean values ± s.d. (n = 12). The asterisks (*) indicate that the expression level of *gtfB*, *gtfC* (at 67 h) is significantly different from other time points (*P*<0.05, Tukey-Krammer HSD). (**B**) This panel displays RT-qPCR analysis of *gtfB*, *gtfC*, and *dexA* gene expression by *S. mutans* in single- or mixed-species biofilm at 67 h, after introduction of 1% (w/v) sucrose. The data shown are mean values ± s.d. (n = 12). The expression level of the specific *S. mutans* genes is significantly different between single- and mixed-species biofilms (*P*<0.05). (**C**) This panel contains MudPIT analysis of expression of EPS-related proteins by *S. mutans* in mixed-species biofilms at specific time points after introduction of 1% (w/v) sucrose (n = 2). Spectral counts were used for quantification of proteins in each of the time points tested; standard deviations for protein data are not shown in the table. (**D**) This panel depicts MudPIT analysis of the abundance of GtfB, GtfC, and DexA by *S. mutans* in single- or mixed-species biofilms at 67 h after introduction of 1% (w/v) sucrose (n = 2). Spectral counts were used for quantification of proteins in each of the time points tested; standard deviations for protein data are not shown in the table.

**Figure 3:**
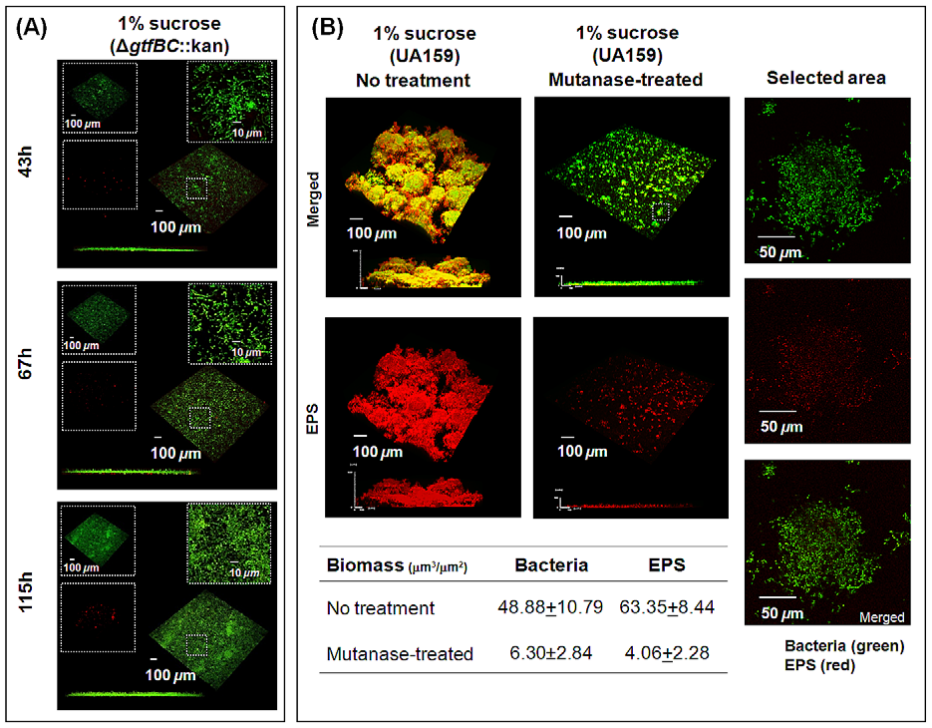
Mixed-species biofilms formed with *S. mutans* Δ*gtfBC*::kan or with parental strain UA159 treated with mutanase. The biofilms were formed in the presence of the *gtfBC* null mutant (A) or parental strain UA159 (B) with 1% (w/v) sucrose. Selected areas in (**A**) and (**B**) show detailed views of separated and merged confocal images of bacterial cells (green) and EPS (red). The biomass values of EPS and bacterial cells in the biofilms were calculated using COMSTAT. The data shown are mean values ± s.d. (n = 15).

The availability of specific carbohydrates also influences the proportion of the bacterial species during mixed-species biofilm development. At the baseline, before introduction of 1% sucrose, bacterial cells on the sHA surface were comprised mainly of *S. oralis* and few *A. naeslundii* cells, with *S. mutans* as the least abundant species (*P*<0.05; Figure 1C). However, the introduction of 1% sucrose (at 29 h) had a dramatic effect. The proportion of *S. mutans* rapidly increased, as it became the dominant species within 115 h-old biofilms (Figure 1B-2; *P*<0.05; CFU values in the figure legend). In contrast, under carbohydrate-limited conditions (0.1% sucrose), the proportions and the number of the bacterial species on the sHA surface were equally distributed (115 h end-point, Figure 1B-1; *P*>0.05). Introduction of 1% glucose (in the absence of sucrose) resulted in a population dominated by *S. oralis* throughout the biofilm development period (Figure 1B-3; *P*<0.05).

As might be expected, the pH in the culture medium surrounding the biofilm also changes over time. The pH values in the culture medium after the introduction of 1% sucrose or 1% glucose were not significantly different from each other (final pH 4.2 to 4.7), but were lower than those in the medium containing 0.1% sucrose (sugar-limiting condition, pH 6.1 to 6.2; *P*<0.05) (see Table S1).

The introduction of 1% sucrose and its subsequent metabolism by *S. mutans* induces the transition from a non-virulent microbial community (high levels of *S. oralis* and small numbers of *S. mutans*; [11], [30]) to a highly acidogenic and aciduric population. The data reveal the importance of EPS production on surfaces, which provides a continuum of adhesion sites for glucan-binding pathogens (e.g. *S. mutans*), while simultaneously generating a matrix with complex 3D architectures that can create uniquely structured low pH environments.

### Dynamics of expression of *S. mutans* EPS-associated genes and proteins

The EPS biogenesis machinery of *S. mutans* (triggered by sucrose) can be affected over time by changes in the environmental conditions and by the presence of other members of the biofilm community [4], [11], [15]. We explored how the introduction of 1% (w/v) sucrose influences the expression of EPS-related genes and proteins in *S. mutans* during mixed-species biofilm formation and maturation. *S. mutans* produces three glucosyltransferases (encoded by *gtfB*, *gtfC* and *gtfD*) and an endodextranase (*dexA*) that participate in the synthesis and degradation of glucans [31], [32]. In addition, a fructosyltransferase and a fructan hydrolase (*ftf* and *fruA*, respectively) [33] catalyze the synthesis and breakdown of fructans composed predominantly of β-2,1 linkages [34].

The expression profiles of the *S. mutans gtfBCD*, *dexA*, *ftf*, and *fruA* genes and their products are shown in Figure 2. The data reveal that the expression of *gtfB*, *gtfC*, and *dexA* was significantly induced between 43 and 67 h (during the transition from initial cell accumulation to EPS-microcolony development on the sHA surface) compared to the level of expression at other time points (e.g. 91 and 115 h) (Figure 2A). MudPIT analyses indicated that the abundance (i.e. spectral counts) of GtfB and GtfC were elevated within the biofilms at 67 h (Figure 2C), showing that greater amounts of these proteins were proportionally present compared to the later time point (115 h). The induction of these exoenzymes at earlier stages of biofilm development is highly relevant for the assembly of the EPS-rich matrix. The secreted Gtfs bind to sHA and bacterial surfaces, displaying enhanced enzymatic activity when bound [4], [17]. The surface-adsorbed GtfB and GtfC enzymes produce large amounts of glucans *in situ*, wherein *S. mutans* binds in high numbers [19]. As a result, it promotes local accumulation of both EPS and bacterial cells.

Biofilms found in nature, such as dental plaque, are usually composed of mixed-species. The influence that one species within a biofilm has on the gene expression of another species within the same biofilm has not been fully explored. We investigated the expression of *gtfB*, *gtfC* and *dexA* in *S. mutans* cells grown in single-species compared to those grown in mixed-species biofilms (after introduction of 1% sucrose). Expression of *S. mutans gtfB* and *gtfC*, and to a lesser extent *dexA*, were elevated in mixed-species biofilms compared to that observed in single-species biofilms (*P*<0.05; Figures 2B and 2D), which resulted in elevated amounts of the respective proteins in the milieu. It is likely that *gtfB* and *gtfC* were induced in the presence of the other oral species by autoinducer molecules, such as Al-2 [35]. The interspecies signaling mediated by the LuxS system (associated with Al-2 synthesis) has been shown to modulate *S. mutans* biofilm formation [36] by enhancing the expression of *gtfB* and *gtfC* but not the *gtfD* gene [37]. This observation is congruent with our gene/protein expression data. Deficient biofilm formation in an *S. mutans luxS* mutant can be complemented by the Al-2 produced by several bacteria *in vitro*, although not by *S. oralis* ATCC 10557 [37] (a different strain, not ATCC 35037, which we used in this study). The exact mechanisms of how Al-2 based signaling up-regulates Gtfs in *S. mutans* within mixed-species biofilms have not been fully elucidated. Whether additional signaling molecules are released by these co-habitants that might suppress *gtfs* expression in *S. mutans* remain to be determined.

Noticeably, the magnitude of the *gtfBC* transcript response was greater than the expected increase in the abundance of the respective proteins. This apparent discrepancy is likely due to differences in the sensitivity of protein (spectral counts by MudPIT) and mRNA transcript (RT-qPCR) detection technologies. In addition, it is possible that post-transcriptional controls can alter protein levels via gene-specific control of translation, or can affect the balance between mRNA stability and degradation [38].

The molecular mechanisms involved with the assembly of the matrix are poorly understood because the basic knowledge about the regulation and temporal production of EPS within mixed-species biofilms are still lacking [5]. We show that the expression of *gtfB* and *gtfC* is enhanced during the early stages of biofilm development and in the presence of other organisms. The synthesis of EPS by these exoenzymes may have direct implications with the sequential construction and the architecture of the matrix.

### The role of GtfB and GtfC in the assembly of structured 3D matrix

The influences of the concerted actions of GtfB and GtfC in the formation and structural organization of biofilm matrix were further explored by means of gene deletion studies. Deletion of both *gtfB* and *gtfC* markedly impaired the ability of the mutant strain to assemble an EPS layer on the sHA surface or further synthesize a 3D EPS-rich matrix. The mutant strain was also unable to form EPS-microcolony complexes on sHA surfaces (vs. mixed-species biofilms formed in the presence of the parental strain *S. mutans* UA159; Figure 3A). Close-up images reveal mostly cell to cell co-aggregation (similar to that observed in 1% glucose group). COMSTAT and DUOSTAT analyses confirmed the absence of any detectable microcolony structures, a reduction in the overall biofilm biomass, and a virtual lack of EPS (data not shown).

The deletion of each of these genes individually resulted in distinctive patterns of EPS and bacterial accumulation on apatitic surfaces (Figure S1). When the *gtfB* null mutant was used to form biofilms, the initial EPS layer was detected, but the assembly of EPS-microcolony complexes was completely abolished (Figure S1). In contrast, when the *gtfC* null mutant was used in the system, the initial EPS-layer formation was greatly impaired while a few small microcolonies (vs. biofilm with UA159) were detected on the sHA surface (possibly associated with still functional *gtfB*).

The surface-formed glucans produced by GtfB (in particular) and GtfC are rich in α-1,3-linked glucose [39], conferring a highly insoluble and structurally rigid polymer that may be critical for the construction of a 3D matrix architecture. We investigated whether digestion of α-1,3 glucosyl-linkages would impact the structural integrity of the EPS-matrix and EPS-microcolony complexes. Introduction of mutanase (α-1,3 glucanase; EC 3.2.1.59), after biofilm formation, essentially caused the collapse of the matrix architecture (Figure 3B). The absence or removal of the insoluble glucans dismantled the framework of the matrix on which cell-associated glucan binding proteins can bind *in situ*. Thus, a lack of insoluble glucan eliminated any structural cell to matrix bridging, causing the disassembly of the EPS-microcolony (see selected area in Figure 3B). COMSTAT analysis also confirmed that there was a substantial reduction in the biomass of both the cellular and EPS components of the biofilms following mutanase treatment (Figure 3B). The data clearly demonstrate that α-1,3-linked glucans (only present in GtfB and to lesser extent in GtfC-derived glucans) are critical for the structural integrity of a 3D matrix and microcolonies; this observation is in agreement with the results derived from the genetic studies.

The location of GtfB- and GtfC-derived polysaccharides may modulate the sequential assembly of the matrix. Each of the secreted Gtfs binds to a particular surface in an enzymatically active form. GtfC has the greatest affinity for the pellicle, whereas GtfB binds preferentially to microbial surfaces [4]. The glucans synthesized by GtfC assemble the initial EPS layers on the sHA surface, which provide enhanced binding sites for *S. mutans* [18], [19], [40]. The highly insoluble, branched, and structurally rigid glucans (rich in α-1,3 linkages) formed by GtfB embed the cells, contributing to the scaffolding of the EPS matrix. In addition, the Gtfs can bind to self-produced glucans, influencing the further growth of the matrix *in situ* [4]. The local accumulation of EPS and bacterial cells mediate the construction of 3D EPS-microcolony complexes through cell-matrix adhesions within mixed-species biofilms. Thus, the enhanced expression of both *gtfB* and *gtfC* by *S. mutans* in the presence of *A. naeslundii* and *S. oralis* (Figure 2B–D) could explain why the size of the microcolonies formed in mixed-species biofilms is larger than those in single-species biofilms (Figure S2).

The mutants lacking the *gtfB* and/or *gtfC* genes are essentially non-virulent *in vivo*, as determined using a well-established rodent model of dental caries [41]. Furthermore, treatment with an egg-derived antibody raised against cell-associated GtfB (that inhibits enzymatic activity) markedly reduced the development of carious lesions in rats [42]. These observations demonstrate the importance of EPS synthesis in the expression of biofilm virulence. However, how this phenomenon is associated with the assembly of the 3D EPS matrix and what its spatial arrangement is with bacterial cells remain to be elucidated. Early studies used only simple cell-labeling methods [4].

### Spatial arrangement between EPS and bacterial cells during the construction of 3D EPS-microcolony complexes

**Figure 4:**
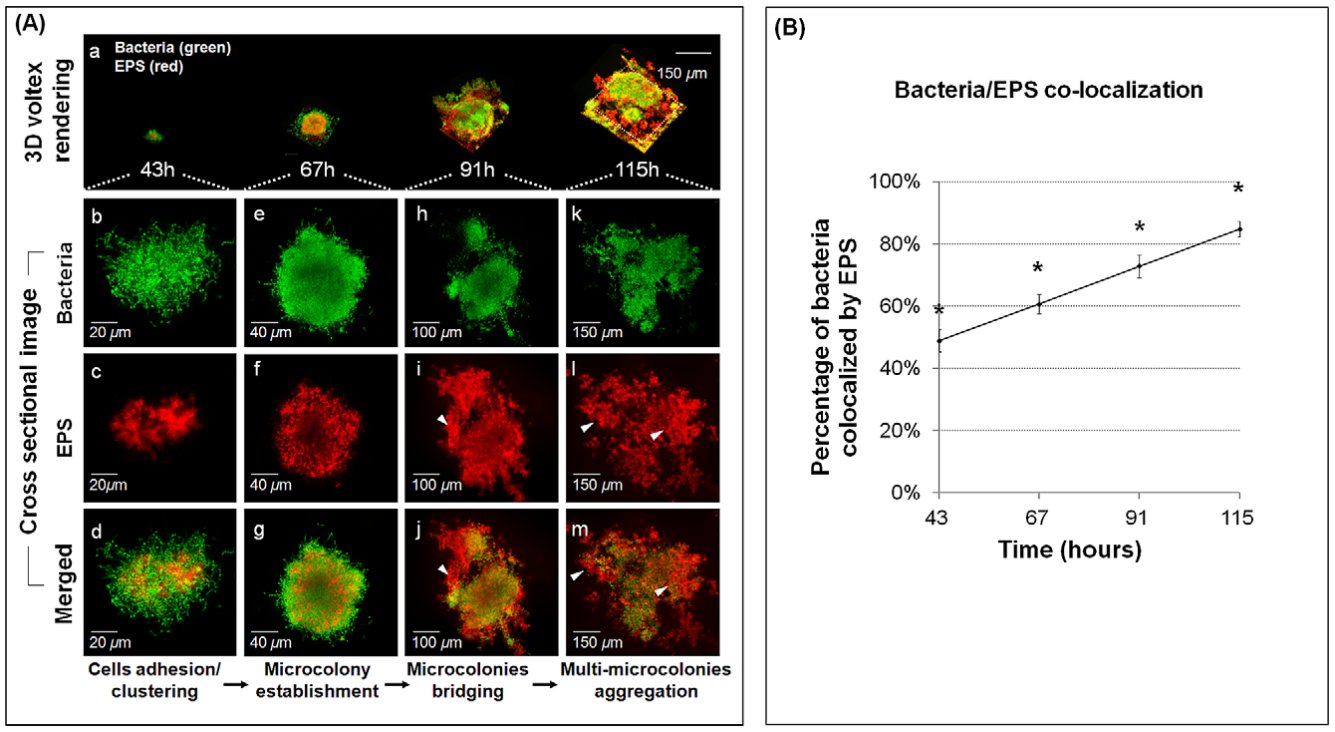
Structural arrangement between EPS and bacterial cells during the assembly of surface-attached EPS-microcolony complex. (**A**) This figure gives representative images of 3D renderings of mixed-species biofilms after introduction of 1% (w/v) sucrose. Panel (a) shows the dynamic evolution of surface-attached microcolonies over time. Panels (b–m) show cross sectional images of selected area for close-up views of the structural organization of EPS (red) and bacterial cells (green) during the development of an EPS-microcolony complex. The arrows indicate EPS bridging (i, j) and providing support for aggregation of multiple microcolonies (l, m). (**B**) The amount of co-localization between bacteria and EPS was calculated using DUOSTAT. The graph shows the percentage of bacteria and EPS colocalized within the biofilm over time (mean values ± s.d; n = 15). The asterisks (*) indicate that the values from each time point are significantly different from each other (*P*<0.05, Tukey-Krammer HSD).

The formation of densely packed bacterial islets is mediated through cell-matrix interactions over time. Therefore, we examined the spatial arrangement of EPS and bacterial cells at each of the developmental stages during morphogenesis of mixed-species biofilms (Figure 4). The same scale was used to demonstrate the three-dimensional evolution of EPS-mediated bacterial aggregation over time (Figure 4A). The cross-sectional images for each time point are shown in Figure 4Ab–m. EPS is detected (1) between the bacterial cells, (2) surrounding, (3) covering, and (4) filling the spaces between the microcolonies, bridging them together and tethering them to the sHA surface.

Based on the location of EPS, we speculate that polysaccharide-mediated biofilm construction forms complex 3D bacterial islets through the following sequence: initial adhesion, cell clustering (Figure 4Ab–d), and formation of a core of EPS-enmeshed bacterial cells that provides a supporting framework to facilitate microcolony establishment (Figure 4e–g). The transition from initial cell clustering to microcolony formation bears some similarities to the transition mediated by PsL (an exopolysaccharide) in *Pseudomonas aeruginosa* biofilms [8]. As the biofilm matures, EPS also surrounds individual microcolonies and appears to bridge them (Figure 4Ah–j), resulting in the formation of multi-microcolony aggregates (Figure 4Ak–m). Results from computational analyses show that >80% of the total cells are co-localized with EPS at 115 h (Figure 4B), as an increasing amount of the bulk of biofilm is composed of polysaccharides (congruent with our confocal images). We suggest that individual microcolonies encased in polysaccharides may serve as architectural units that become connected during biofilm construction, forming compartmentalized networks that confer highly heterogeneous yet cohesive environments within the 3D architecture.

The adhesion between the bacterial cell and the EPS-matrix may be mediated in part through cell-surface glucan-binding protein C (GbpC) and possibly GbpB, both of which are expressed by *S. mutans* [4], [43]. Secreted (extracellular) GbpA and GbpD may also bind to and provide cross-linking between glucans on the sHA surface and those on bacterial surfaces [4], [43], thus contributing to the maintenance of the 3D architecture. Furthermore, extracellular DNA (eDNA) may have a structural function in the extracellular matrix. eDNA enhances *S. mutans* adhesion and surface aggregation [44] and could be incorporated during EPS-rich matrix development by binding to bacterial cells and to exopolysaccharides [23], possibly contributing to the formation of microcolonies.

The assembly of EPS-matrix changes the biofilm architecture in 3D while the bacterial populations are dynamically shifted towards dominance of *S. mutans*, a Gtf-producing, glucan-binding and aciduric/acidogenic pathogen. Concomitantly, EPS-microcolony complexes are formed in specific regions within the biofilms (bound to the sHA surface and tethered to the matrix) via localized cell to matrix adhesions, while other areas are devoid of such structures. This highly heterogeneous architecture and assembly of a variety of bacterial islets, particularly those in close proximity to sHA, may create ecological and environmental niches that are essential for biofilm virulence.

### Relevance of biofilm-matrix architecture in virulence

Acid production alone may not be the key factor in the expression of virulence, but rather how and where acidic microenvironments are formed and maintained within the biofilm. Results from previous studies reveal average pH values in biofilms. In contrast, our data show punctate areas of low pH values that are heterogeneously distributed within complex 3D biofilm architecture.

#### Creation of localized acidic microenvironment

**Figure 5:**
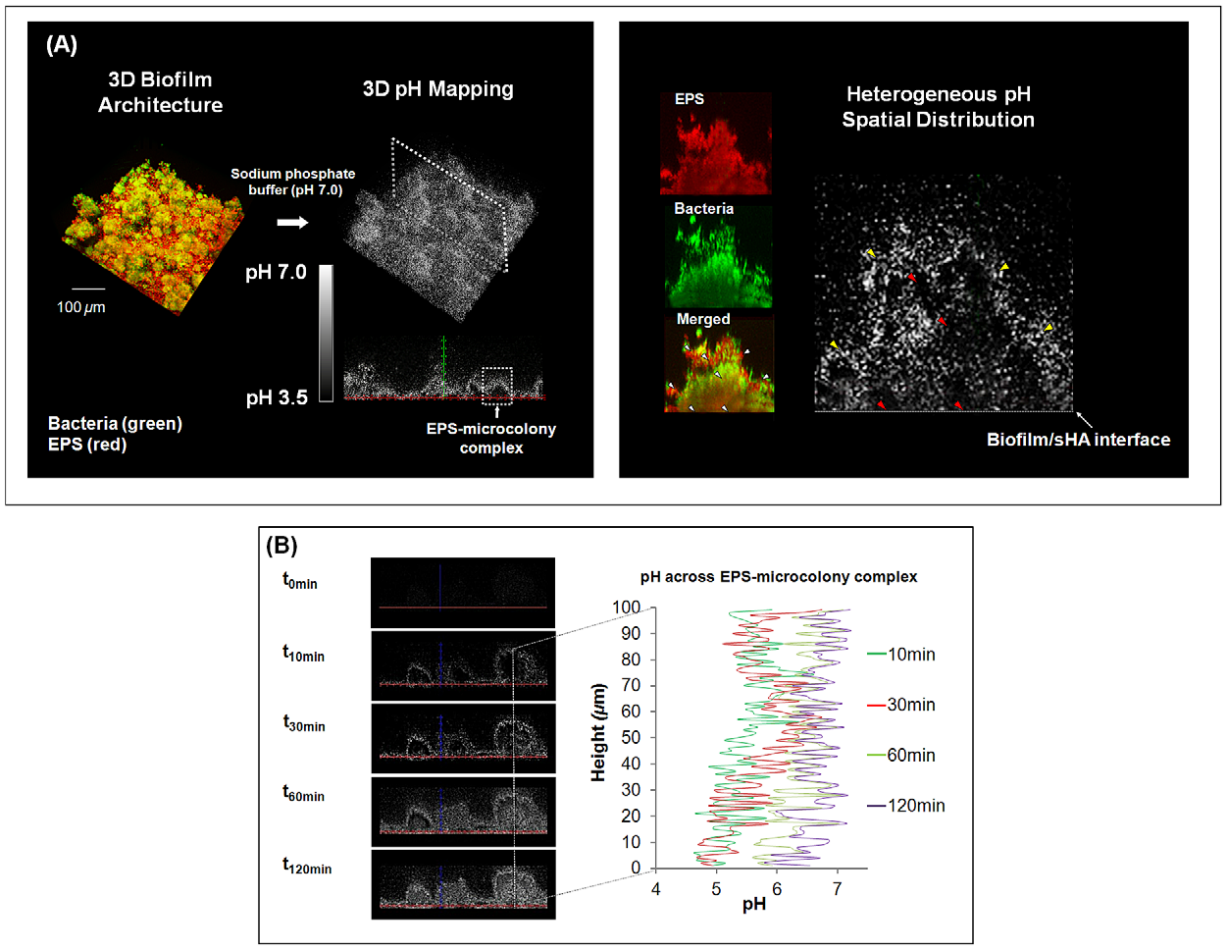
Three-dimensional pH mapping of intact mixed-species biofilm. (**A**) This figure displays representative images of the pH profile throughout the biofilm 3D architecture and the spatial distribution of pH within the selected EPS-microcolony complex in mixed-species biofilms formed after introduction of 1% sucrose. Dark areas are indicative of regions of low pH, while white or light areas are indicative of regions of pH that are close to neutral, as indicated by the scale bar. The EPS channel is depicted in red, while the cells are depicted in green. Arrows indicate various acidic pH regions within the microcolony and at the sHA interface. Red arrows indicate acidic pH regions (i) across the structure and (ii) at the microcolony/sHA interface. Yellow arrows indicate pH close to neutral at the microcolony/fluid phase interface. White arrows indicate the corresponding pH landmarks in the merged panel. (**B**) This panel shows temporal changes of *in situ* pH across the EPS-microcolony complex after the biofilm was exposed to sodium phosphate-based buffer (pH 7.0) for a total of 120 min.

The *in situ* pH mapping of the biofilm shows spatially heterogeneous pH microenvironments throughout the 3D biofilm architecture, despite the presence of buffered neutral pH in the external environment (Figure 5A). The darker areas in the pH profile represent acidic pH values, whereas the brighter areas represent pH values that are close to neutral (indicated by the pH-visualization bar in Figure 5A).

Environments with low pH values were found only in the interior of the EPS-microcolony complexes within the biofilm (highlighted in Figure 5A). A close-up view of the pH map of these bacterial islets reveals three distinct pH profiles (see arrows; Figure 5A) as follows: (1) regions that display pH values that are close to neutral at the microcolony/fluid phase, (2) a range of acidic “pockets” across the structure (from the sHA surface to the fluid phase interface), and (3) acidic pH at the microcolony/sHA interface. The presence of discrete regions containing low pH (4.5 to 5.5) indicates that the acids accumulated and confined in these particular areas are not rapidly neutralized when incubated in neutral pH (7.0) buffer; more than 120 minutes of exposure are required before neutralization occurs (Figure 5B).

**Figure 6:**
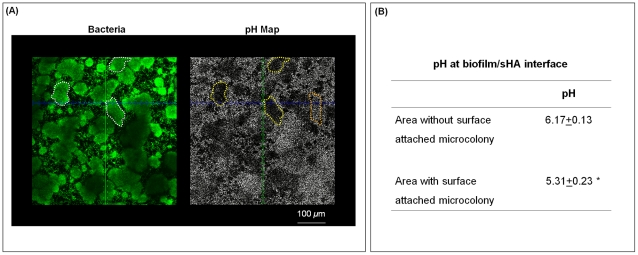
*In situ* pH mapping at the EPS-microcolony complex and sHA interface. (**A**) This figure shows representative cross sectional images (bird's eye view) at the sHA surface. White marks indicate the areas with surface-attached microcolonies (in the bacteria channel; depicted in green) while yellow marks show the corresponding area in the pH channel; the orange mark indicate an area without surface-attached microcolony. Dark areas are indicative of regions of low pH, while white or light areas are indicative of regions of pH that are close to neutral. (**B**) This table shows the pH values at the sHA interface for areas with and without surface-attached microcolonies after 30 min incubation in sodium phosphate-based buffer (pH 7.0). The data shown are mean values ± s.d. (n = 90). The asterisks (*) indicate that the differences between the two conditions are statistically significant (*P*<0.05, Tukey-Krammer HSD).

The presence of acidic regions at the microcolony-sHA interface has direct implications for cariogenesis. Acids present at the surface of the biofilm attachment increase local demineralization of the hydroxyapatite mineral, which is the main constituent of the tooth enamel [45]. Therefore, cross-sectional images of the biofilm at the sHA surface and pH mapping in the same field of view were acquired to illustrate the spatial distribution of surface-attached microcolonies and pH values (Figure 6A). It is readily apparent that the presence of surface attached-microcolonies (white line) corresponded with acidic pH on the sHA surface (darker areas highlighted with yellow lines). The pH measurements taken by ratiometric imaging analysis show that the pH at the microcolony/sHA interface was below 5.5 (regarded as the critical pH for rapid dissolution of enamel), and the pH value was significantly more acidic compared to areas without such particular 3D structures (highlighted in orange in the pH map image) (*P*<0.05; Figure 6B).

**Figure 7:**
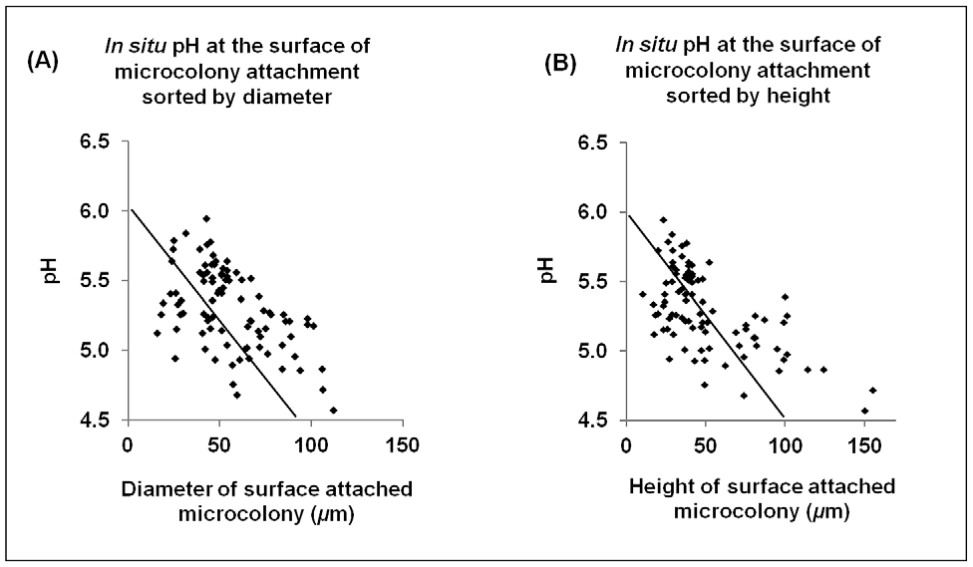
Relationship of *in situ* pH at sHA surface with size of surface-attached EPS-microcolony complexes. This figure depicts mixed-species biofilms (67 h) formed after the introduction of 1% (w/v) sucrose, which were processed for measurement of *in situ* pH at the interface between sHA surface and microcolonies. All the pH values were sorted by (**A**) the diameter and (**B**) height (distance from surface to fluid-phase) of the surface-attached microcolonies. The data shown are values from three separate experiments (n = 90). Correlation analysis showed that there is a linear relationship between pH and microcolony diameter (Pearson's test, *P*<0.0001, r-square 0.259) and height (*P*<0.0001, r-square 0.367).

The presence of localized and elevated concentrations of acids at the surface of biofilm attachment has clinical relevance, as it could explain the heterogeneous pattern of carious lesions during the onset of this disease in humans. Dental caries is characterized by the temporal and localized/punctate appearance of regions of demineralization on the tooth enamel under plaque, clinically known as “white spots” [46]. These “white spots” eventually coalesce into a broader lesion, rather than a diffuse and homogeneous layer of erosion. Thus, the appearance of the lesions and the level of demineralization may be associated with the size of the surface-attached EPS-microcolony complexes. Correlation analysis showed that there is a linear relationship between low pH on the sHA surface and the diameter or height of these bacterial islets (Figure 7).

Biofilms formed with the *gtfB* null mutant (lacking a 3D matrix and EPS-microcolony complexes) were readily neutralized after incubation in neutral pH buffer and failed to generate any acidic microenvironments (Figure S3). Interestingly, biofilms formed with the *gtfC* null mutant contained isolated and small EPS-microcolony complexes, which displayed some acidic pockets, although these were neutralized within 30 minutes (Figure S3). Collectively, the data clearly demonstrate that there is a substantial limitation to diffusion, which is associated with the assembly of EPS-rich matrix and the size of EPS-enmeshed microcolonies. Their presence could trap acid at the sHA surface and/or restrict access of neutralizing buffer (e.g. saliva) to areas of low pH within the biofilm. The data are consistent with Tatevossian's findings [47], which show that the concentration of solutes in human dental plaque fluid differs markedly from those observed in saliva.

The precise mechanisms involved in limiting diffusion are unclear. It is possible that the presence of glucans produced by oral streptococci limit diffusion of charged ions in and out of the plaque-biofilm, whereas uncharged substances, such as sucrose, may diffuse readily [45], [47], [48]. In contrast, others have noted that EPS has little effect on acid diffusion when using artificial plaque and *in vitro* modeling [49], [50]. However, none of these studies evaluated intact biofilms, as investigated here. Our data show the importance of how the matrix is assembled three-dimensionally and how it is spatially arranged with the bacterial cells to act as a diffusion-limiting physical barrier.

The presence of a high density of acidogenic and aciduric bacteria within these structures can facilitate the conversion of sucrose (and other fermentable carbohydrates) into acids via the glycolytic pathway. Furthermore, degradation of extracellular soluble glucans by *S. mutans* dextranase (also up-regulated within biofilm) and degradation of intracellular polysaccharides increases the availability of fermentable sugars (e.g. maltose and glucose) [4], [23], [31]. The metabolism of these carbohydrates could also contribute to the acidification of the resultant 3D milieu. The presence of alkali-generating bacteria in biofilms may also influence the local pH [51]. The use of metabolic activity markers could help to elucidate whether acidification is an active process or a secondary consequence of cell numbers (density). It is apparent that the combination of the physical and metabolic factors could, at least in part, explain the spatial distribution of punctate acidic regions in our *in vitro* biofilms and the low fasting pH values observed in plaque from caries-active individuals.

The formation of a heterogeneous acidic environment is biologically relevant, as pH changes could trigger dynamic shifts in the microbial population according to the ecological plaque model [11]. Niches with low pH values would activate the acid stress adaptive responses of particular organisms clustered in these EPS-sheltered acidic pockets, favoring survival/growth of aciduric and glucan-binding pathogens, such as *S. mutans* (initially in low-numbers) [24], [25]. Local pH concentrations would also affect the spatial distribution of the bacterial species throughout the 3D biofilm architecture. The positioning of the microorganisms and their stress response mechanisms may correlate with their susceptibility to, or affinity for, low pH environments. In addition, persistent low pH niches may also affect the expression of the *gtf* genes, as expression of *gtfBC* is induced in response to acidification and the presence of excess metabolizable carbohydrates [52]. These genes would therefore likely be up-regulated locally, coinciding with areas that are undergoing active matrix remodeling and acidogenesis.

#### Creation of protective microenvironments

The microorganisms within biofilms display increased resistance to antimicrobials in comparison to their planktonic counterparts [1], [5], [53]. This phenomenon is generally associated with poor antimicrobial penetration (due to the presence of an extracellular matrix), activation of adaptive stress responses (including production of persister cells and induction of quorum sensing mechanisms), and physiological heterogeneity within the biofilm population [26], [54]. However, the importance of spatially heterogeneous environments and spatially complex cell-matrix arrangements in bacterial antimicrobial resistance within biofilms has received limited attention.

**Figure 8:**
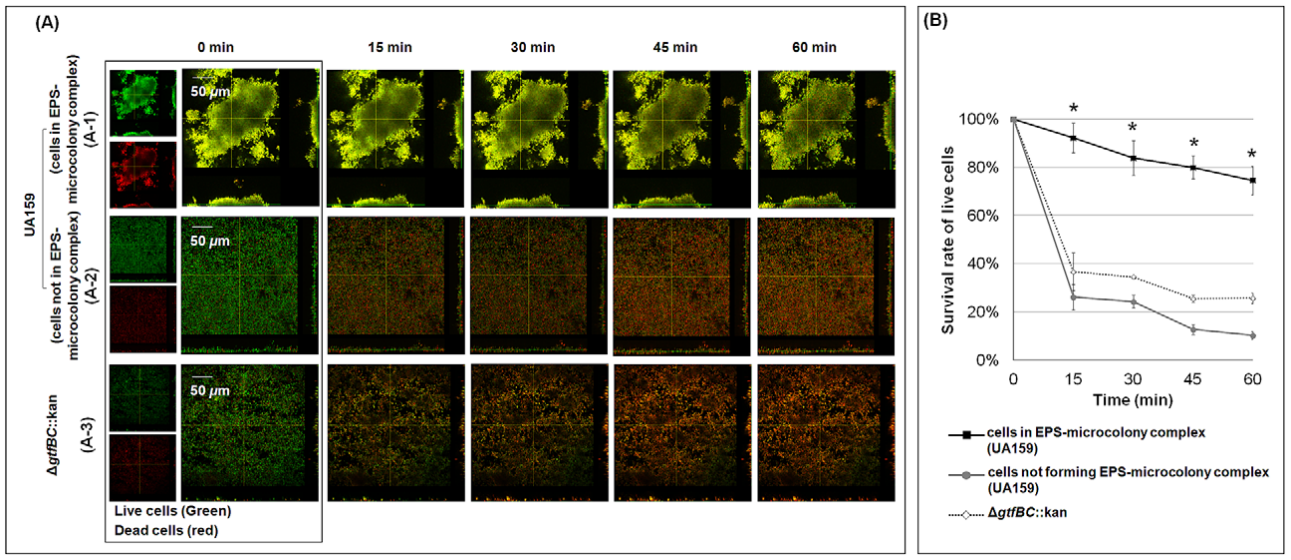
Time-lapse imaging of bacterial viability after exposure of the biofilm to 0.12% (v/v) chlorhexidine (CHX). Panel (**A**) shows the relative populations of live (SYTO 9-labeled; depicted in green) and dead (propidium iodide-labeled; depicted in red) cells over time in the mixed-species biofilm formed with the parental strain (A-1; cells within EPS-microcolony complex and A-2; cells outside EPS-microcolony complex) and the Δ*gtfBC*::kan mutant (A-3) after exposure to CHX. Panel (**B**) shows the survival rate of live cells. The data shown are mean values ± s.d. (n = 6). The asterisks (*) indicate that the differences between the conditions tested (cells within EPS-microcolony complex vs. cells outside EPS-microcolony complex or cells not forming such structures, i.e. biofilm formed with Δ*gtfBC*::kan) are statistically significant (*P*<0.05).

The construction of bacterial islets that are enmeshed and surrounded by an EPS-rich matrix could create localized areas of microbial resistance to antimicrobials within the biofilm. Therefore, we evaluated the viability of two distinct groups of bacterial cells within the same intact biofilm system in response to the immediate local effects of chlorhexidine (CHX) exposure (by time-lapsed measurements). We examined (1) cells within EPS-microcolony complexes, and (2) cells outside (or not forming) such structures (Figure 8). CHX is a broad spectrum antibacterial agent commonly used in mouthwash (at concentrations between 0.12–0.2%) to suppress the levels of mutans streptococci in the oral cavity [55]. The overlay images of live (in green) and dead (in red) cells after exposure to 0.12% CHX are shown in Figure 8A. Clearly, the inside of the EPS-enmeshed microcolony (Figure 8A-1) contains a greater proportion of live cells than those residing outside the structured communities over time (Figure 8A-2).

The survival rate of each group of bacterial cells was also calculated using the confocal images (Figure 8B). The data show that less than 10% of the total cells within the EPS-microcolony complex were killed after 15 min exposure. In contrast, more than 70% of the cells outside the structure were not viable (*P*<0.05). In the absence of the matrix, cells were highly susceptible to CHX. Mixed-species biofilms formed with the *gtfBC* null mutant strain were rapidly killed by CHX (Figure 8A-3; Figure 8B). Similarly, bacterial cells that generally do not organize into EPS-microcolony complexes, such as those in biofilms grown in 1% glucose, were also highly susceptible to killing by CHX (60–75% of the cells were dead after 15 min exposure to CHX; data not shown). Chlorhexidine (CHX) is a cationic bisbiguanidine [55]. The presence of negatively charged EPS (possibly due to association with lipoteichoic acids) could retard the diffusion of positively charged CHX molecules into biofilms [48], [53], [56]. However, our data show that antimicrobial resistance is not a general attribute associated with the simple physical presence of EPS, but rather with the way the matrix is assembled with the bacterial cells, which likely restricts access of CHX into EPS-microcolony structures.

Furthermore, the cells clustered within EPS-microcolony complexes may develop direct antimicrobial resistance in response to cell density and/or the presence of acidic microenvironments in these bacterial islets [26], [54]. These observations support the concept that antimicrobial resistance is a site-specific property within the greater biofilm architecture, although we were unable to explore the additional possibilities described above.

Results from a recent study [57] demonstrated the importance of a structured environment (using fabricated interconnected chambers within a microfluidic device) for rapid evolution of bacterial antibiotic resistance via the creation of steep chemical gradients. Our findings agree with the modeling of Zhang et al. [57], recapitulating spatial heterogeneities in our *de facto* biofilm system. Simply put, the EPS-matrix creates highly compartmentalized, yet interconnected and cohesive microenvironments. As a result, acidic regions and niches of antimicrobial resistance are created locally, demonstrating the importance of the 3D architecture in the evolution of virulent oral biofilms.

### Conclusion

In this study, we show that the concerted actions of *S. mutans*-derived GtfB and GtfC, in the presence of sucrose, form a heterogeneous layer of EPS on the sHA surface. The surface-formed EPS promotes local accumulation of bacteria. The continuous production of EPS *in situ* coats the organisms and they rapidly aggregate, forming densely packed bacterial islets (termed EPS-microcolony complexes). Over time, these 3D structures become interconnected through surrounding EPS. The sequential accumulation of EPS on sHA and bacterial surfaces assembles an intricate and complex 3D matrix that leads to a highly compartmentalized architecture within an intact mixed-species biofilm.

The relevance of the matrix 3D architecture in biofilm virulence is revealed by our novel pH mapping approach, which uses a pH marker incorporated directly into the matrix. We illustrate spatially heterogeneous pH microenvironments manifested by “acidic pockets” found in the interior of the EPS-enmeshed bacterial islets only, despite exposure to a neutral buffer. This observation is clinically relevant in the context of the oral cavity in which saliva at neutral pH is abundant. The low pH niches favor acid-tolerant organisms, ensuring continued localized acid production; an EPS-rich environment promotes accumulation of the glucan-binding pathogen *S. mutans*. The environmental changes, associated with the matrix architecture, shift the microbial balance from a non-virulent to a virulent condition. Moreover, regions of acidic pH (<5.5) were found only in the areas where surface-attached EPS-enmeshed microcolonies were present on the sHA surface. The presence of an elevated concentration of acids at the apatitic surface favors local demineralization (while preventing remineralization), thereby facilitating development of carious lesions at these sites. Furthermore, the bacterial cells residing within EPS-microcolony complexes throughout the biofilm architecture are locally sheltered against a potent antimicrobial agent (CHX).

We demonstrated that *S. mutans* does not always need to dominate within plaque to influence virulence, because the Gtfs released, even at the time of biofilm initiation, adhere to the pellicle and other organisms, mediating the construction of the EPS-rich matrix. Moreover, the expression of GtfB and GtfC is enhanced in the presence of other oral bacteria. Results from previous studies have linked Gtfs to biofilm formation (using standard genetic and/or simple cell staining assays in mono-species biofilms) and expression of virulence (using rodent models) [4]. Our data illuminate the functional roles of GtfB- and GtfC-derived EPS in the basic biology of biofilms. We show how the assembly of a 3D EPS-matrix architecture helps to create spatial heterogeneities, and facilitates the formation of a variety of structured acidic microenvironments within a mixed-species biofilm. Clearly, the data from our study offer new avenues for further elucidation of how cell to matrix interactions govern the creation of ecological and environmental niches that are essential for expression of virulence by an oral biofilm.

The information generated here may pave the way for designing future studies aiming to develop novel methods that better prevent the formation and/or promote the disruption of virulent dental plaque. Furthermore, we introduce novel approaches to study the dynamics of biofilm construction and heterogeneity within the intact 3D architecture. Our observations have broad applicability beyond the mouth, because the imaging techniques introduced could be adapted for use in other biofilm systems in which polymers are formed during matrix development, and varied microenvironments are created.

## Materials and Methods

### Bacterial strains

*Streptococcus mutans* UA159 serotype c (ATCC 700610), a proven virulent cariogenic dental pathogen selected for genomic sequencing [58], *Actinomyces naeslundii* ATCC 12104, and *Streptococcus oralis* ATCC 35037 were used to generate mixed-species biofilms. We selected *S. oralis* and *A. naeslundii* in addition to *S. mutans* because these three organisms are found in the supragingival plaque of humans [59]. *S. oralis* binds to the pellicle and is one of the most commonly detected pioneer colonizers of the saliva-coated tooth surface [59], [60] in the absence of sucrose; strain ATCC 35037 also produces soluble glucans from sucrose and is acid-tolerant [60]. *A. naeslundii* is also detected during the early stages of plaque formation and may be associated with development of root caries; strain ATCC 12104 is acidogenic and produces fructans [61].

Furthermore, *gtfB*, *gtfC*, and *gtfBC* null mutants (Δ*gtfB*::kan, Δ*gtfC*::kan, and Δ*gtfBC*::kan, respectively), constructed from the parental *S. mutans* UA159 strain, were generated by standard allelic replacement with a non-polar kanamycin resistance marker and verified by DNA sequence analysis. These strains were kindly provided by Dr. Robert A. Burne (Department of Oral Biology, University of Florida, Gainesville, Florida) [22]. The strains were checked via PCR before use. The cultures were stored at −80°C in tryptic soy broth containing 20% glycerol.

### Mixed-species biofilm model

The biofilm method is based on a batch culture approach using saliva-coated hydroxyapatite (sHA) discs, which was designed to mimic the formation of biofilms according to the “ecological plaque-biofilm” concept [11], as recently described by Koo et al. [22]. *S. mutans* UA159, *A. naeslundii* ATCC 12104, and *S. oralis* ATCC 35037 cells were grown in ultrafiltered (10 kDa molecular-weight cut-off membrane; Prep/Scale, Millipore, MA) buffered tryptone-yeast extract broth (UFTYE; 2.5% tryptone and 1.5% yeast extract, pH 7.0) with 1% glucose at 37°C and 5% CO_2_ to mid-exponential phase; the ultrafiltration process removes high-molecular weight mannans and other polysaccharides that could interfere with our analyses. These bacterial suspensions were then mixed to provide an inoculum with a defined microbial population of *S. mutans* (10^2^ CFU/ml), *A. naeslundii* (10^6^ CFU/ml), and *S. oralis* (10^7^ CFU/ml), which are critical for the reproducibility of our model [22]. Biofilms were formed on hydroxyapatite discs (1.25 cm diameter, surface area of 2.7±0.2 cm^2^, Clarkson Chromatography Products, Inc., South Williamsport, PA) coated with filter-sterilized clarified whole saliva [22], which were placed in a vertical position using a custom-made disc holder (Figure S4). The mixed population of *S. mutans*, *A. naeslundii*, and *S. oralis* was inoculated in 2.8 ml of UFTYE with 0.1% (w/v) sucrose, and incubated at 37°C and 5% CO_2_. During the first 19 h, the organisms were grown undisturbed to allow initial biofilm formation. At this point (19 h), the culture medium was replaced with fresh medium and the biofilms were grown until 29 h for establishment of the initial biofilm community [22]. At 29 h of biofilm growth, the biofilms were transferred to UFTYE containing 1% (w/v) sucrose or 1% (w/v) glucose to induce environmental changes to simulate a cariogenic challenge, while an additional set of biofilms was grown in UFTYE with 0.1% sucrose. The culture medium was then replaced twice daily (8 am and 6 pm) until the end of the experimental period (115 h). The pH of spent culture medium was measured daily using a standard pH electrode. The biofilms were analyzed at specific time points (29, 43, 67, 91, and/or 115 h) by means of confocal imaging/fluorescence, biochemical, reverse transcription quantitative PCR (RT-qPCR), and multi-dimensional protein identification technology (MudPIT) assays (see experimental design scheme in Figure S4). Furthermore, mixed-species biofilms were also formed in the presence of *S. mutans* UA159 mutant strains defective in *gtfB* and/or *gtfC* because they are critical for insoluble EPS synthesis and expression of virulence *in vivo* [4], [41], [62]. The *gtfD* mutant was not included because it did not have significant effect on matrix formation and biofilm architecture. All the *gtf* mutants used in this study exhibited similar planktonic growth rates (vs. UA159) as monitored (at OD_600 nm_) using an automated turbidimetric Bioscreen C MBC system (Oy Growth Curves Ab Ltd, Helsinki, Finland).

### Laser scanning confocal fluorescence microscopy (LCSFM) imaging of biofilm matrix

The sequential assembly of the matrix was determined by directly incorporating a fluorescent marker during synthesis of the extracellular polysaccharide (EPS)-matrix, allowing examination of the three-dimensional (3D) structure within intact biofilms [63], [64]. Briefly, 1 µM Alexa Fluor 647-labeled dextran conjugate (molecular weight, 10 kDa; absorbance/fluorescence emission maxima of 647/668 nm; Molecular Probes, Invitrogen Corp., Carlsbad, CA) was added to the culture medium from the beginning of and during the development of the biofilms. This technique is based on the observation that (fluorescently-labeled) dextran serves as a primer and acceptor for Gtfs (particularly GtfB), and is incorporated into newly formed glucan by the exoenzyme during synthesis of the EPS-matrix over the course of biofilm development [63]; it does not stain the bacterial cells at the concentration used in this study [63]. All the bacterial species in the biofilms were labeled by means of SYTO 9 green fluorescent nucleic acid stain (485/498 nm; Molecular Probes) using standard protocols [63], [64]. The imaging was performed using an Olympus FV 1000 two photon laser scanning microscope (Olympus, Tokyo, Japan) equipped with a 10× (0.45 numerical aperture) or 25× LPlan N (1.05 numerical aperture) water immersion objective lens. The excitation wavelength was 810 nm, and the emission wavelength filter for SYTO 9 was a 495/540 OlyMPFC1 filter, while the filter for Alexa Fluor 647 was an HQ655/40M-2P filter [22]. Each biofilm was scanned at 5 positions randomly selected at the microscope stage [65], and confocal image series were generated by optical sectioning at each of these positions. Three independent biofilm experiments were performed, and 10 image stacks (512×512 pixel for quantification or 1024×1024 pixel for visualization in tagged image file format) were collected for each experiment.

### Computational analyses of the confocal biofilm images

The confocal images were analyzed using software for simultaneous visualization and quantification of EPS and bacterial cells within intact biofilms [64]. Amira 5.0.2 (Mercury Computer Systems Inc., Chelmsford, MS) was used to create 3D renderings of each structural component (EPS and bacteria) of the biofilms for visualization of the morphology and 3D architecture as detailed previously [64], [65]. COMSTAT and newly developed DUOSTAT (both are available as free downloadable software at http://www.imageanalysis.dk) were used for quantitative analysis. COMSTAT and DUOSTAT, written as scripts for MATLAB 5.3 (The Mathworks, Natick, MA, USA) equipped with the Image Processing Toolbox (see details at http://www.imageanalysis.dk), converts pixels from confocal image stacks into numerical values, facilitating quantitative characterization of each structural component within 3D biofilm images [64]. We used COMSTAT to calculate the biomass, number, and size (volume, diameter, and height) of microcolonies, as detailed elsewhere [65]. DUOSTAT was developed to calculate the co-localization of two biofilm components (e.g. EPS and bacterial cells) throughout the 3D biofilm architecture [64]. The software overlays the confocal image data sets (one for total bacteria and another for EPS) from the same biofilm, and calculates the proportion of total bacteria image pixels that are co-localized with EPS [64].

### Non-invasive 3D *in situ* pH measurement and visualization

The ability to characterize microenvironments (e.g. spatial pH distribution) within biofilms without disrupting their architectural integrity is a major technical challenge. We used a fluorescent pH indicator, Lysosensor yellow/blue (Molecular Probes), to determine the *in situ* pH in our biofilm model. Lysosensor yellow/blue is conjugated with dextran (10 kDa MW), which allows the labeled dextran to be directly incorporated into the EPS-matrix [63], as described previously. Once incorporated into the biofilm matrix, the pH values can be measured based on fluorescence intensity ratios of the dual-wavelength fluorophore [66]. Lysosensor yellow/blue exhibits a dual-emission spectral peak (fluorescence emission maxima 452 nm and 521 nm) that is pH dependent [66]. The pH measurements are based on the principle that protonation of the fluorophore shifts its emission spectra, such that increasing ion concentrations increase fluorescence intensity at one wavelength, while decreasing it at the other [67]. The fluorescence intensity of both emission wavelengths and the ratio of fluorescent intensity (I450/I520) within each biofilm image was measured using Image J 1.44 and its calculation tools (e.g. channel 1 divide by channel 2 as detailed in http://rsbweb.nih.gov/ij/download.html). Titration curves of ratios versus pH (ranging from 3.5 to 7.0) were calculated as described previously [67], and were used to convert Lysosensor yellow/blue emission (fluorescence intensity) ratios to pH values. The intensity ratio of both wavelengths serve as a quantitative measure of pH, and Lysosensor yellow/blue is particularly suitable for acidic environments (low sensitivity maximum; pKa∼4.2), as expected in our biofilm model [66]. The experimental protocol and titration curve of ratios versus pH are provided in the supporting information (Protocol S1 and Figure S5).

The confocal images of Lysosensor-incorporated biofilms were acquired before and after incubation in Na_2_HPO_4-_citric acid buffer at pH 7.0. The images were acquired after 10, 30, 60, and 120 min of incubation in the neutral buffer to evaluate the spatial distribution of pH throughout the intact 3D biofilm architecture. For pH measurement, the ratio of fluorescence intensity of selected areas within each biofilm image were converted to pH values using the titration curves (as described in Protocol S1 and Figure S5) and Image J software (http://www.uhnresearch.ca/facilities/wcif/PDF/ImageJ_Manual.pdf). For visualization of *in situ* pH distribution within the 3D biofilm architecture, the fluorescence intensity ratios (corresponding to the pH values between 7.0 and 3.5) of all confocal images were reconstructed using Image J, then Amira. The fluorescence intensity was converted into grayscale using the Amira tool-box to correlate with the pH range from 7.0 (white) to 3.5 (black) (Protocol S1). Unlabeled biofilms were also imaged to check whether the autofluorescence of each biological component of the biofilm (i.e. bacterial cells and glucans) would interfere with pH quantification at the wavelengths and laser intensity used in our study; there was no interference with the measurements based on our image analysis.

### Microbiological analysis of the mixed-species bacterial population

The biofilms were removed at specific time points (Figure S4) and homogenized by sonication in sterile 0.89% (w/v) NaCl solution (30 sec pulse at an output of 7W; Branson Sonifier 150, Branson Ultrasonics, Danbury, CT); the sonication procedure does not kill any of the bacterial species used in this study [22]. The homogenized suspension was used to determine the number of viable cells by plating on blood agar using an automated EddyJet Spiral Plater (IUL, SA, Barcelona, Spain) to determine the number of viable cells (total number of colony-forming units per biofilm). The three species were differentiated by observation of colony morphology in conjunction with microscopic examination of cells from selected colonies [68]. In addition, the bacterial populations were also assessed by qPCR using species specific primers for *S. mutans* [69], *A. naeslundii*, and *S. oralis*. The *A. naeslundii* (5- CCTCTGGCTTAACTGGGG -3 and 5- CATTCCACCGCTACACCA -3) and *S. oralis* (5- CCGCATAAGAGTAGATGTTG -3 and 5- GCCTTGGTGAGCCGTTAC -3) species-specific primers were designed using Beacon Designer 2.0 software (Premier Biosoft International, Palo Alto, CA, USA). Bacterial cell quantification using standard curves based on the genome size of each organism was performed as described previously [69]. The data was expressed as total numbers, and as proportion of each of the bacterial species within the biofilm.

### *S. mutans* gene expression analysis using multiplex RT-qPCR

We performed RT-qPCR to measure the expression profiles of specific genes directly associated with extracellular polysaccharide matrix development, including *gtfB*, *gtfC*, *gtfD*, *fruA*, *ftf*, and *dexA*. RNA was extracted and purified using protocols optimized for biofilms [70]. The RNA integrity number (RIN) for all of our samples was ≥9.0, as determined by on-chip capillary electrophoresis with the Agilent 2100 Bioanalyzer (Agilent Technologies, Inc., Santa Clara, CA). Briefly, cDNAs were synthesized using 0.5 µg of purified RNA and the BioRad iScript cDNA synthesis kit (Bio-Rad Laboratories, Inc., Hercules, CA). To check for DNA contamination, purified total RNA without reverse transcriptase served as a negative control. The resulting cDNA and negative controls were amplified by a Bio-Rad CFX96 system (Bio-Rad Laboratories, Inc., CA) using specific primers and TaqMan probes (Table S2) and iQ Multiplex Powermix (Bio-Rad Laboratories). A standard curve was plotted for each primer set as detailed elsewhere [71]. The standard curves were used to transform the critical threshold cycle (Ct) values to relative numbers of cDNA molecules. Comparative expression was calculated by normalizing each gene of interest to the 16S rRNA gene, which served as a reference gene [71].

### *S. mutans* protein expression analysis by MudPIT

We also examined whether changes in *S. mutans* gene expression would reflect the levels of their corresponding protein products in biofilms. The *S. mutans* protein expression profile in whole biofilms was analyzed by MudPIT and specific database search algorithms described previously [72]–[75]. Briefly, biofilms at 67 and 115 h of growth were homogenized by sonication (30 sec pulse at an output of 7W; Branson Sonifier 150) in the presence of protease cocktail inhibitor (Roche). After homogenization, aliquots were taken for bacteria quantification by qPCR and total protein quantification (by acid digestion followed by ninhydrin assay [76]); the protease cocktail inhibitor did not affect the ninhydrin assay as determined experimentally. Total protein was extracted, precipitated, and digested with trypsin, as detailed elsewhere [73], [74]. MS/MS spectra were searched with the ProLuCID algorithm [75] or the Sequest algorithm [77] against an NCBI-RefSeq *S. mutans* and *S. oralis* database (01/01/2010) concatenated to a decoy database in which the sequence for each entry in the original database was reversed [73]. All searches were parallelized and performed on a Beowulf computer cluster consisting of 100 1.2 GHz Athlon CPUs. ProLuCID results were assembled and filtered using the DTASelect (version 2.0) program [78]. The false positive rates are estimated by the program from the number and quality of spectral matches to the decoy database. We focused on proteins encoded by *S. mutans* UA159, and compared biofilms at 67 h- vs. 115 h-old. The level of protein abundance in mixed-species was calculated by normalizing the spectral count to the relative numbers of *S. mutans* by multiplying the spectral count values by the ratio of *S. mutans* CFU to the total CFU in the mixed-species biofilms samples.

### Susceptibility of EPS matrix to mutanase

The EPS matrix formed in mixed-species biofilms was examined for susceptibility to mutanase [α-1,3 glucanase; EC 3.2.1.59], produced from *Trichoderma harzianum*, as detailed elsewhere [39]. The enzyme (Novo Nordisk, Denmark) was kindly provided by Dr. William H. Bowen (Center for Oral Biology, University of Rochester Medical Center). Biofilm was formed on the sHA surface, and the EPS-matrix was labeled with Alexa Fluor 647-labeled dextran conjugate (Molecular Probes) as described in the previous sections. At 91 h of biofilm development, mutanase (2 units of enzyme) were added to the culture media. At 115 h, biofilms were retrieved and the bacteria cells were labeled with SYTO 9 (Molecular Probes). Biofilms were examined using confocal fluorescence imaging, and subjected to AMIRA/COMSTAT analysis.

### Cell viability within intact biofilms by time-lapsed killing assay

The effects of chlorhexidine (CHX) on the viability of biofilm cells immediately after introduction of the antimicrobial agent were assessed by time-lapsed measurements. The intact biofilms (formed with parental UA159 strain or *gtfBC* null mutant) were dip washed twice in 0.89% NaCl, then transferred to a Petri dish (Diameter 3.5 cm) containing 2.5 µM SYTO 9 (for labeling live-cells; Molecular Probes) and 2.5 µM propidium iodide-PI (for labeling dead-cells; Molecular Probes) in 4 ml of 0.89% NaCl, and incubated for 30 min at room temperature using standard protocols described by Hope and Wilson [53]. After labeling, confocal images were acquired using an Olympus FV 1000 two photon laser scanning microscope with an 25× LPlan N (1.05 numerical aperture) water-immersion objective lens (Olympus). The excitation wavelength was set at 780 nm and the emission filter for SYTO 9 was the 495/540 OlyMPFC1 filter, while PI was the HQ655/40M-2P filter. Immediately after completion of the first scan, and while keeping the biofilm in the same position, 48 µl of 10% (v/v) CHX solution was carefully added to the 4 ml cell culture dish and mixed to yield a final concentration of 0.12% CHX (v/v). CHX does not react with the dyes used in this study [53]. For the time-lapse series, images of the same field of view were taken at 15, 30, 45, and 60 minutes after CHX exposure. The total biomass of live and dead bacterial cells in each series of confocal images was quantified using COMSTAT. The ratio of live to the total bacteria at each time point was calculated, and the survival rate of live cells (relative to live cells prior to exposure to CHX) was plotted. The survival rate was calculated based on the amount of viable cells over time. The initial number of viable cells immediately before the introduction of CHX (time point 0 min; t _0 min._) was considered to be 100%. The number of viable cells after the introduction of CHX was then determined at each time point and the percent-survival rate was determined by comparing to t _0 min_. Thus, the influence of the structural organization of the matrix on the killing effects of CHX can be monitored *in situ* and in real-time.

### Statistical analysis

An exploratory data analysis was performed to determine the most appropriate statistical test; the assumptions of equality of variances and normal distribution of errors were also checked. The data were then analyzed using ANOVA, and the F test was used to test any difference between the groups (different experimental conditions and distinct time-points within same experimental condition). When significant differences were detected, a pairwise comparison was made between all the groups using Tukey-Kramer HSD method to adjust for multiple comparisons. Pearson's test was also used to evaluate the linear correlation between pH value and microcolony height (or diameter). Software JMP version 3.1 (SAS Institute, Cary, NC, USA) was used to perform the statistical analyses. The level of significance was set at 5%.

## Supporting Information

Figure S1 **Representative 3D rendering of mixed-species biofilms formed in the presence of** ***S. mutans*** **UA159 mutant strains.** (A) Δ*gtfB*::kan or (B) Δ*gtfC*::kan after introduction of 1% (w/v) sucrose.
(TIF)

Figure S2 **Comparison of biofilm architecture by** ***S. mutans*** **alone (single-species) or in the presence of other species (mixed-species) after introduction of 1% sucrose.** (A) Representative 3D images of biofilms (and microcolonies) formed by *S. mutans* alone or mixed with other species; (B) number and volume of microcolonies as determined using COMSTAT (n = 10).
(TIF)

Figure S3 **Mapping of** ***in situ*** **pH of intact mixed-species biofilm formed in the presence of** ***S. mutans*** **UA159 mutant strains.** (A) Δ*gtfB*::kan or (B) Δ*gtfC*::kan after introduction of 1% (w/v) sucrose.
(TIF)

Figure S4 **Overview of the experimental design of the mixed-species biofilm model.**
(TIF)

Figure S5 **Titration curve used to convert Lysosensor yellow/blue emission (fluorescence intensity) ratios to pH values.**
(TIF)

Protocol S1 **Detailed methodology for 3D in situ pH mapping.**
(DOC)

Table S1 **pH of the culture medium during mixed-species biofilm formation.**
(DOC)

Table S2 **Primers and TaqMan probes used for multiplex RT-qPCR assays.**
(DOC)

## References

[1] Stoodley P, Sauer K, Davies DG, Costerton JW (2002). Biofilms as complex differentiated communities.. Annu Rev Microbiol.

[2] Foster JS, Palmer RJ, Kolenbrander PE (2003). Human oral cavity as a model for the study of genome-genome interactions.. Biol Bull.

[3] Dye BA, Tan S, Smith V, Lewis BG, Barker LK (2007). Trends in oral health status: United States, 1988–1994 and 1999–2004.. Vital Health Stat.

[4] Bowen WH, Koo H (2011). Biology of Streptococcus mutans-Derived Glucosyltransferases: Role in Extracellular Matrix Formation of Cariogenic Biofilms.. Caries Res.

[5] Flemming HC, Wingender J (2010). The biofilm matrix.. Nat Rev Microbiol.

[6] Branda SS, Vik S, Friedman L, Kolter R (2005). Biofilms: the matrix revisited.. Trends Microbiol.

[7] Karatan E, Watnick P (2009). Signals, regulatory networks, and materials that build and break bacterial biofilms.. Microbiol Mol Biol Rev.

[8] Ma L, Conover M, Lu H, Parsek MR, Bayles K (2009). Assembly and development of the Pseudomonas aeruginosa biofilm matrix.. PLoS Pathog.

[9] Mann EE, Rice KC, Boles BR, Endres JL, Ranjit D (2009). Modulation of eDNA release and degradation affects Staphylococcus aureus biofilm maturation.. PLoS One.

[10] Dewhirst FE, Chen T, Izard J, Paster BJ, Tanner AC (2010). The human oral microbiome.. J Bacteriol.

[11] Marsh PD (2003). Are dental diseases examples of ecological catastrophes?. Microbiology.

[12] Nobbs AH, Lamont RJ, Jenkinson HF (2009). Streptococcus adherence and colonization.. Microbiol Mol Biol Rev.

[13] Gross EL, Leys EJ, Gasparovich SR, Firestone ND, Schwartzbaum JA (2010). Bacterial 16S sequence analysis of severe caries in young permanent teeth.. J Clin Microbiol.

[14] Palmer CA, Kent R, Loo CY, Hughes CV, Stutius E (2010). Diet and caries-associated bacteria in severe early childhood caries.. J Dent Res.

[15] Burne RA (1998). Oral streptococci… products of their environment.. J Dent Res.

[16] Rolla G, Ciardi JE, Schultz SA (1983). Adsorption of glucosyltransferase to saliva coated hydroxyapatite. Possible mechanism for sucrose dependent bacterial colonization of teeth.. Scand J Dent Res.

[17] Vacca-Smith AM, Bowen WH (1998). Binding properties of streptococcal glucosyltransferases for hydroxyapatite, saliva-coated hydroxyapatite, and bacterial surfaces.. Arch Oral Biol.

[18] Schilling KM, Bowen WH (1992). Glucans synthesized in situ in experimental salivary pellicle function as specific binding sites for Streptococcus mutans.. Infect Immun.

[19] Venkitaraman AR, Vacca-Smith AM, Kopec LK, Bowen WH (1995). Characterization of glucosyltransferaseB, GtfC, and GtfD in solution and on the surface of hydroxyapatite.. J Dent Res.

[20] Hamada S, Tai S, Slade HD (1978). Binding of glucosyltransferase and glucan synthesis by Streptococcus mutans and other bacteria.. Infect Immun.

[21] McCabe RM, Donkersloot JA (1977). Adherence of Veillonella species mediated by extracellular glucosyltransferase from Streptococcus salivarius.. Infect Immun.

[22] Koo H, Xiao J, Klein MI, Jeon JG (2010). Exopolysaccharides produced by Streptococcus mutans glucosyltransferases modulate the establishment of microcolonies within multispecies biofilms.. J Bacteriol.

[23] Klein MI, DeBaz L, Agidi S, Lee H, Xie G (2010). Dynamics of Streptococcus mutans transcriptome in response to starch and sucrose during biofilm development.. PLoS One.

[24] Lemos JA, Burne RA (2008). A model of efficiency: stress tolerance by Streptococcus mutans.. Microbiology.

[25] Quivey RG, Kuhnert WL, Hahn K (2000). Adaptation of oral streptococci to low pH.. Adv Microb Physiol.

[26] Stewart PS, Franklin MJ (2008). Physiological heterogeneity in biofilms.. Nat Rev Microbiol.

[27] Reese S, Guggenheim B (2007). A novel TEM contrasting technique for extracellular polysaccharides in in vitro biofilms.. Microsc Res Tech.

[28] Rani SA, Pitts B, Beyenal H, Veluchamy RA, Lewandowski Z (2007). Spatial patterns of DNA replication, protein synthesis, and oxygen concentration within bacterial biofilms reveal diverse physiological states.. J Bacteriol.

[29] Werner E, Roe F, Bugnicourt A, Franklin MJ, Heydorn A (2004). Stratified growth in Pseudomonas aeruginosa biofilms.. Appl Environ Microbiol.

[30] Takahashi N, Nyvad B (2011). The role of bacteria in the caries process: ecological perspectives.. J Dent Res.

[31] Hayacibara MF, Koo H, Vacca-Smith AM, Kopec LK, Scott-Anne K (2004). The influence of mutanase and dextranase on the production and structure of glucans synthesized by streptococcal glucosyltransferases.. Carbohydr Res.

[32] Guggenheim B, Burckhardt JJ (1974). Isolation and properties of a dextranase from streptococcus mutans OMZ 176.. Helv Odontol Acta.

[33] Burne RA, Penders JE (1992). Characterization of the Streptococcus mutans GS-5 fruA gene encoding exo-beta-D-fructosidase.. Infect Immun.

[34] Ebisu S, Kato K, Kotani S, Misaki A (1975). Structural differences in fructans elaborated by streptococcus mutans and Strep. salivarius.. J Biochem.

[35] Waters CM, Bassler BL (2005). Quorum sensing: cell-to-cell communication in bacteria.. Annu Rev Cell Dev Biol.

[36] Wen ZT, Yates D, Ahn SJ, Burne RA (2010). Biofilm formation and virulence expression by Streptococcus mutans are altered when grown in dual-species model.. BMC Microbiol.

[37] Yoshida A, Ansai T, Takehara T, Kuramitsu HK (2005). LuxS-based signaling affects Streptococcus mutans biofilm formation.. Appl Environ Microbiol.

[38] Le Roch KG, Johnson JR, Florens L, Zhou Y, Santrosyan A (2004). Global analysis of transcript and protein levels across the Plasmodium falciparum life cycle.. Genome Res.

[39] Kopec LK, Vacca-Smith AM, Bowen WH (1997). Structural aspects of glucans formed in solution and on the surface of hydroxyapatite.. Glycobiology.

[40] Cross SE, Kreth J, Zhu L, Sullivan R, Shi W (2007). Nanomechanical properties of glucans and associated cell-surface adhesion of Streptococcus mutans probed by atomic force microscopy under in situ conditions.. Microbiology.

[41] Yamashita Y, Bowen WH, Burne RA, Kuramitsu HK (1993). Role of the Streptococcus mutans gtf genes in caries induction in the specific-pathogen-free rat model.. Infect Immun.

[42] Kruger C, Pearson SK, Kodama Y, Vacca Smith A, Bowen WH (2004). The effects of egg-derived antibodies to glucosyltransferases on dental caries in rats.. Caries Res.

[43] Lynch DJ, Fountain TL, Mazurkiewicz JE, Banas JA (2007). Glucan-binding proteins are essential for shaping Streptococcus mutans biofilm architecture.. FEMS Microbiol Lett.

[44] Das T, Sharma PK, Busscher HJ, van der Mei HC, Krom BP (2010). Role of extracellular DNA in initial bacterial adhesion and surface aggregation.. Appl Environ Microbiol.

[45] Zero DT, van Houte J, Russo J (1986). The intra-oral effect on enamel demineralization of extracellular matrix material synthesized from sucrose by Streptococcus mutans.. J Dent Res.

[46] Nikiforuk G, Nikiforuk G (1985). The Caries Process - Morphological and Chemical Events.. Understanding Dental Caries: Etiology and mechanisms, basic and clinical aspects.

[47] Tatevossian A (1990). Fluoride in dental plaque and its effects.. J Dent Res.

[48] Melvaer KL, Helgeland K, Rolla G (1974). A charged component in purified polysaccharide preparations from Streptococcus mutans and Streptococcus sanguis.. Arch Oral Biol.

[49] Dibdin GH, Shellis RP (1988). Physical and biochemical studies of Streptococcus mutans sediments suggest new factors linking the cariogenicity of plaque with its extracellular polysaccharide content.. J Dent Res.

[50] Hata S, Mayanagi H (2003). Acid diffusion through extracellular polysaccharides produced by various mutants of Streptococcus mutans.. Arch Oral Biol.

[51] Burne RA, Marquis RE (2000). Alkali production by oral bacteria and protection against dental caries.. FEMS Microbiol Lett.

[52] Li Y, Burne RA (2001). Regulation of the gtfBC and ftf genes of Streptococcus mutans in biofilms in response to pH and carbohydrate.. Microbiology.

[53] Hope CK, Wilson M (2004). Analysis of the effects of chlorhexidine on oral biofilm vitality and structure based on viability profiling and an indicator of membrane integrity.. Antimicrob Agents Chemother.

[54] Lewis K (2001). Riddle of biofilm resistance.. Antimicrob Agents Chemother.

[55] Jones CG (1997). Chlorhexidine: is it still the gold standard?. Periodontol 2000.

[56] Rolla G, Oppermann RV, Bowen WH, Ciardi JE, Knox KW (1980). High amounts of lipoteichoic acid in sucrose-induced plaque in vivo.. Caries Res.

[57] Zhang Q, Lambert G, Liao D, Kim H, Robin K (2011). Acceleration of emergence of bacterial antibiotic resistance in connected microenvironments.. Science.

[58] Ajdic D, McShan WM, McLaughlin RE, Savic G, Chang J (2002). Genome sequence of Streptococcus mutans UA159, a cariogenic dental pathogen.. Proc Natl Acad Sci U S A.

[59] Beighton D (2005). The complex oral microflora of high-risk individuals and groups and its role in the caries process.. Community Dent Oral Epidemiol.

[60] Fujiwara T, Hoshino T, Ooshima T, Sobue S, Hamada S (2000). Purification, characterization, and molecular analysis of the gene encoding glucosyltransferase from Streptococcus oralis.. Infect Immun.

[61] Bergeron LJ, Morou-Bermudez E, Burne RA (2000). Characterization of the fructosyltransferase gene of Actinomyces naeslundii WVU45.. J Bacteriol.

[62] Tanzer JM, Freedman ML, Fitzgerald RJ, Rosan SEMaB (1985). Virulence of mutants defective in glucosyltransferase, dextran-mediated aggregation, or dextranase activity.. Molecular Basis of Oral Microbial Adhesion.

[63] Klein MI, Duarte S, Xiao J, Mitra S, Foster TH (2009). Structural and molecular basis of the role of starch and sucrose in Streptococcus mutans biofilm development.. Appl Environ Microbiol.

[64] Klein MI, Xiao J, Heydorn A, Koo H (2011). An analytical tool-box for comprehensive biochemical, structural and transcriptome evaluation of oral biofilms mediated by mutans streptococci.. J Vis Exp.

[65] Xiao J, Koo H (2010). Structural organization and dynamics of exopolysaccharide matrix and microcolonies formation by Streptococcus mutans in biofilms.. J Appl Microbiol.

[66] DePedro HM, Urayama P (2009). Using LysoSensor Yellow/Blue DND-160 to sense acidic pH under high hydrostatic pressures.. Anal Biochem.

[67] Hunter RC, Beveridge TJ (2005). Application of a pH-sensitive fluoroprobe (C-SNARF-4) for pH microenvironment analysis in Pseudomonas aeruginosa biofilms.. Appl Environ Microbiol.

[68] Guggenheim B, Giertsen E, Schupbach P, Shapiro S (2001). Validation of an in vitro biofilm model of supragingival plaque.. J Dent Res.

[69] Catalan MA, Scott-Anne K, Klein MI, Koo H, Bowen WH (2011). Elevated incidence of dental caries in a mouse model of cystic fibrosis.. PLoS One.

[70] Cury JA, Koo H (2007). Extraction and purification of total RNA from Streptococcus mutans biofilms.. Anal Biochem.

[71] Koo H, Seils J, Abranches J, Burne RA, Bowen WH (2006). Influence of apigenin on gtf gene expression in Streptococcus mutans UA159.. Antimicrob Agents Chemother.

[72] Liu H, Sadygov RG, Yates JR (2004). A model for random sampling and estimation of relative protein abundance in shotgun proteomics.. Anal Chem.

[73] Peng J, Elias JE, Thoreen CC, Licklider LJ, Gygi SP (2003). Evaluation of multidimensional chromatography coupled with tandem mass spectrometry (LC/LC-MS/MS) for large-scale protein analysis: the yeast proteome.. J Proteome Res.

[74] Washburn MP, Wolters D, Yates JR (2001). Large-scale analysis of the yeast proteome by multidimensional protein identification technology.. Nat Biotechnol.

[75] Xu T, Venable JD, Park SK, Cociorva D, Lu B (2006). ProLuCID, a fast and sensitive tandem mass spectra-based protein identification program.. Mol Cell Proteomics.

[76] Moore S, Stein WH (1954). A modified ninhydrin reagent for the photometric determination of amino acids and related compounds.. J Biol Chem.

[77] Eng JK, McCormack AL, Yates JR (1994). An Approach to Correlate Tandem Mass Spectral Data of Peptides with Amino Acid Sequences in a Protein Database.. J Am Soc Mass Spectrom.

[78] Cociorva D, D LT, Yates JR (2007). Validation of tandem mass spectrometry database search results using DTASelect.. Curr Protoc Bioinformatics Chapter.

