# Supplementary figures and images for: The Exopolysaccharide Matrix Modulates the Interaction between 3D Architecture and Virulence of a Mixed-Species Oral Biofilm
Source: PLoS Pathog. 2012 Apr 5;8(4):e1002623. doi: 10.1371/journal.ppat.1002623 (PMC3320608; doi:10.1371/journal.ppat.1002623)

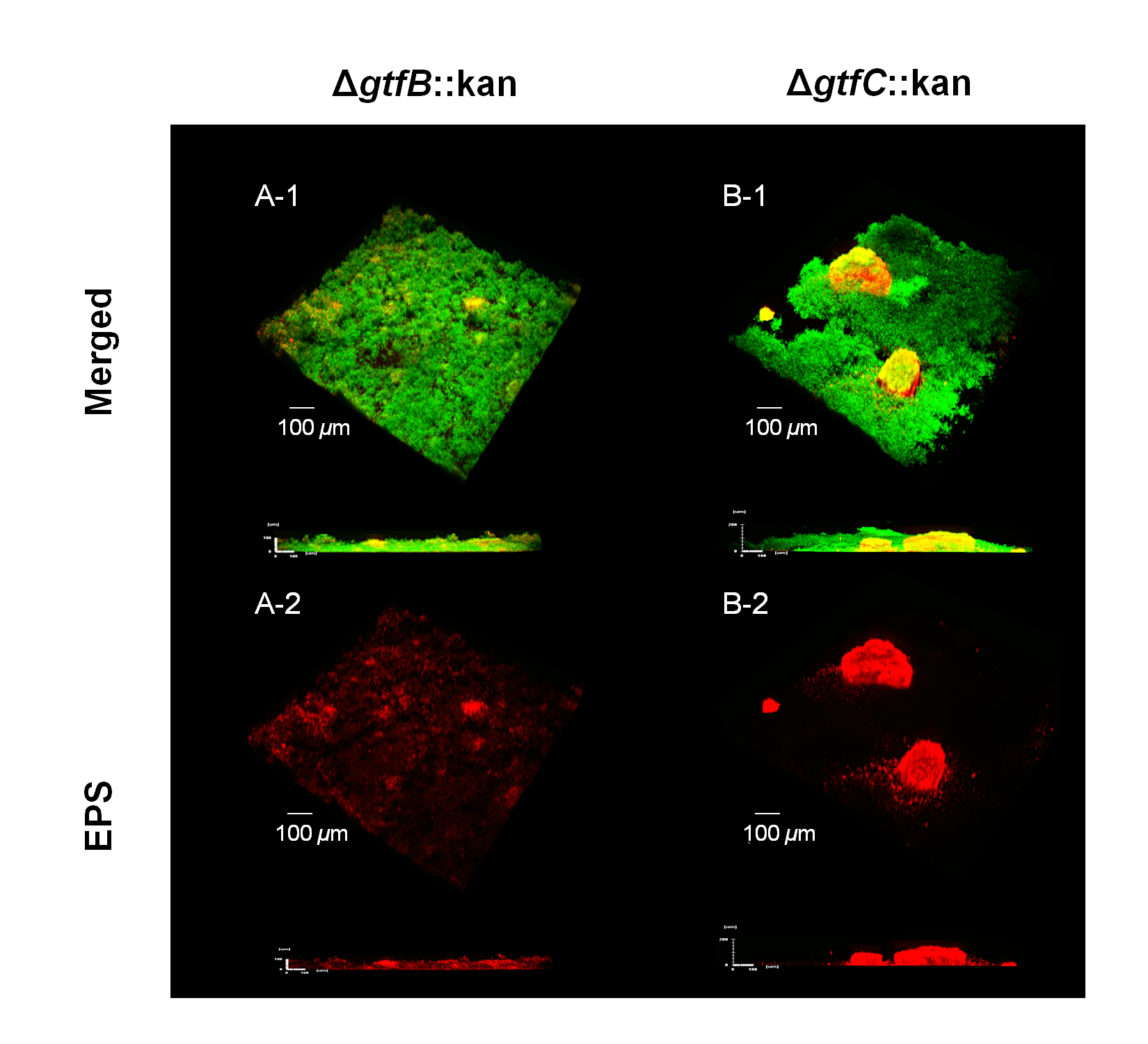

Supplement: Figure S1 — Representative 3D rendering of mixed-species biofilms formed in the presence of S. mutans UA159 mutant strains. (A) ΔgtfB::kan or (B) ΔgtfC::kan after introduction of 1% (w/v) sucrose. (TIF) [file ppat.1002623.s001.tif]

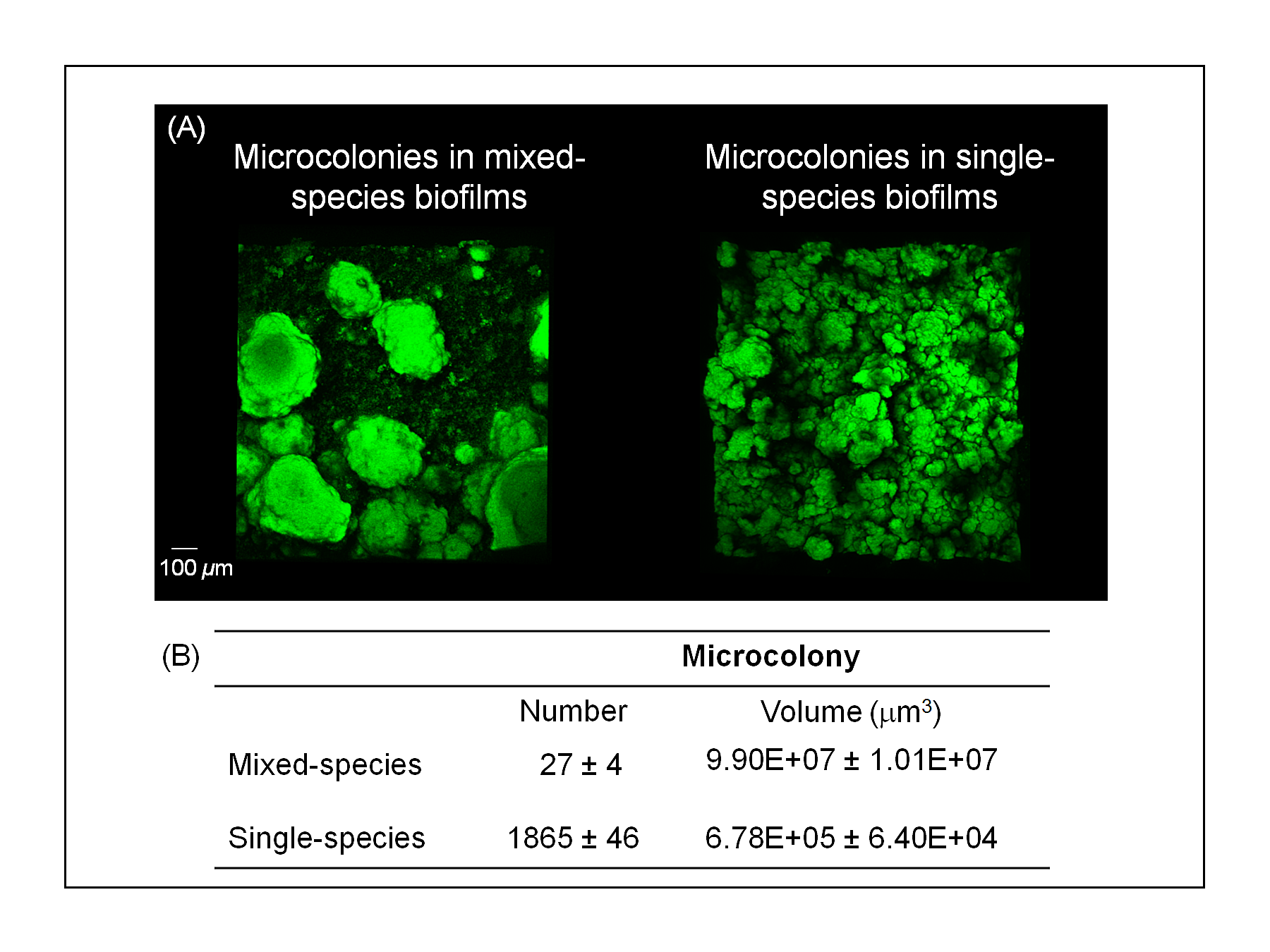

Supplement: Figure S2 — Comparison of biofilm architecture by S. mutans alone (single-species) or in the presence of other species (mixed-species) after introduction of 1% sucrose. (A) Representative 3D images of biofilms (and microcolonies) formed by S. mutans alone or mixed with other species; (B) number and volume of microcolonies as determined using COMSTAT (n = 10). (TIF) [file ppat.1002623.s002.tif]

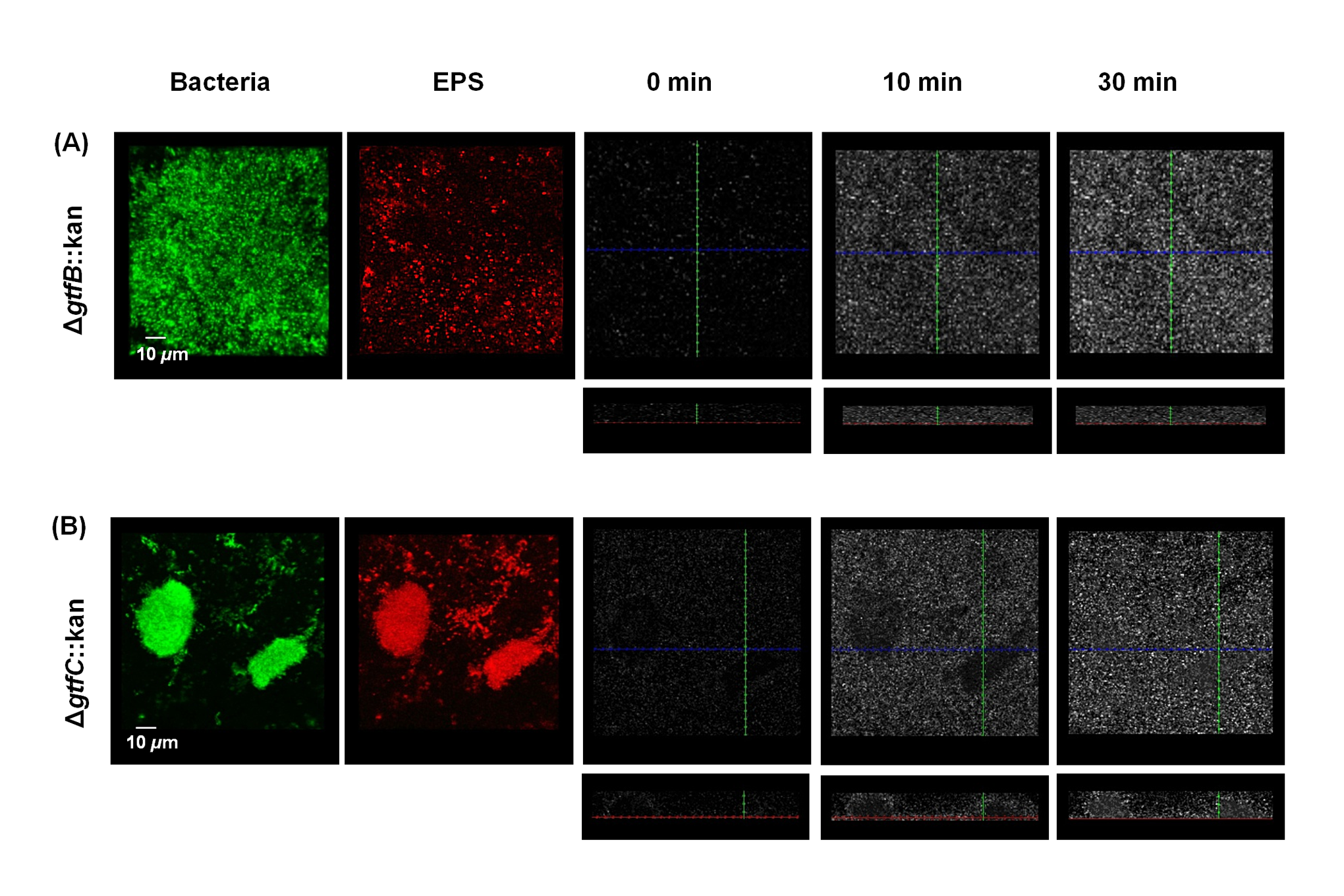

Supplement: Figure S3 — Mapping of in situ pH of intact mixed-species biofilm formed in the presence of S. mutans UA159 mutant strains. (A) ΔgtfB::kan or (B) ΔgtfC::kan after introduction of 1% (w/v) sucrose. (TIF) [file ppat.1002623.s003.tif]

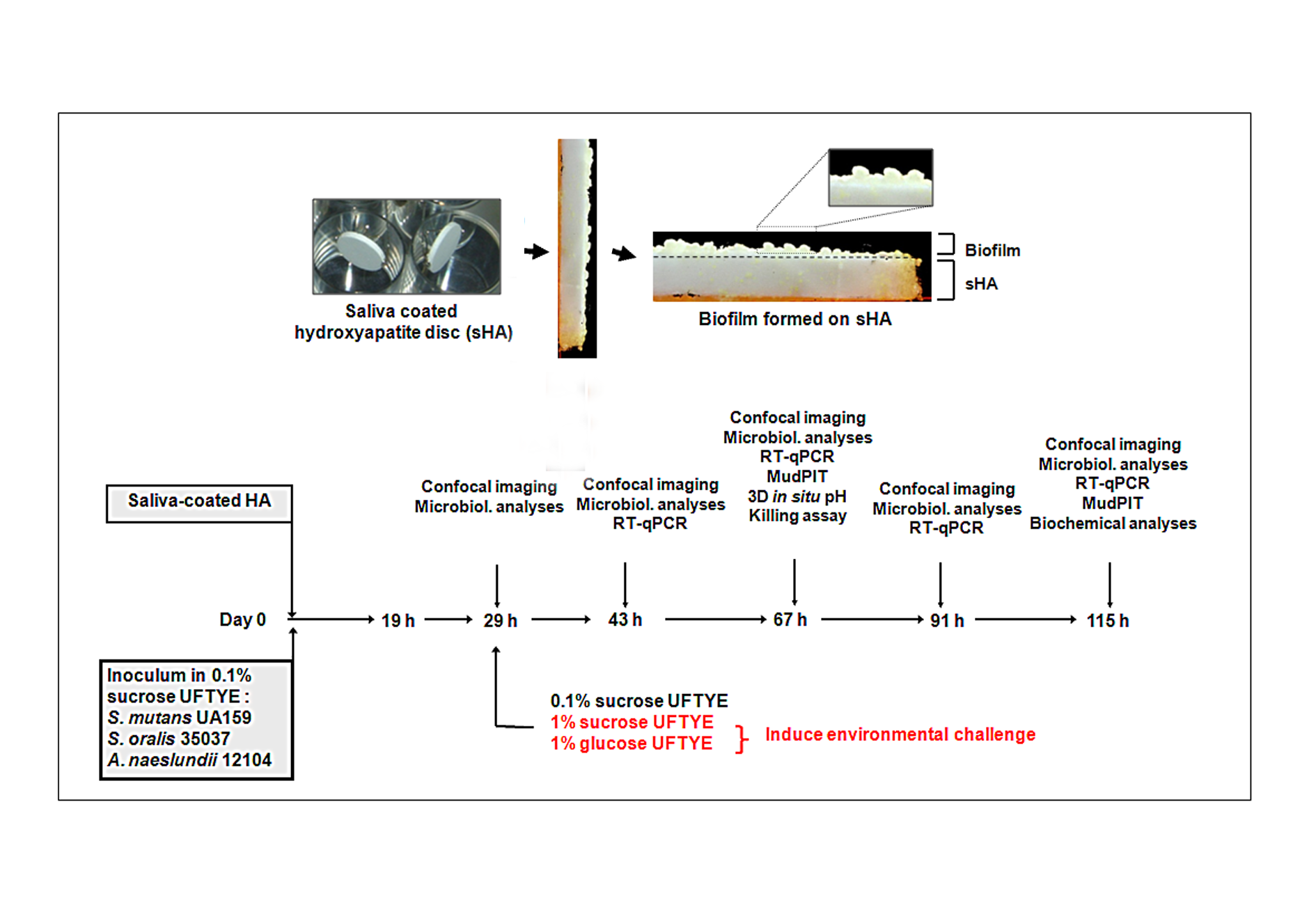

Supplement: Figure S4 — Overview of the experimental design of the mixed-species biofilm model. (TIF) [file ppat.1002623.s004.tif]

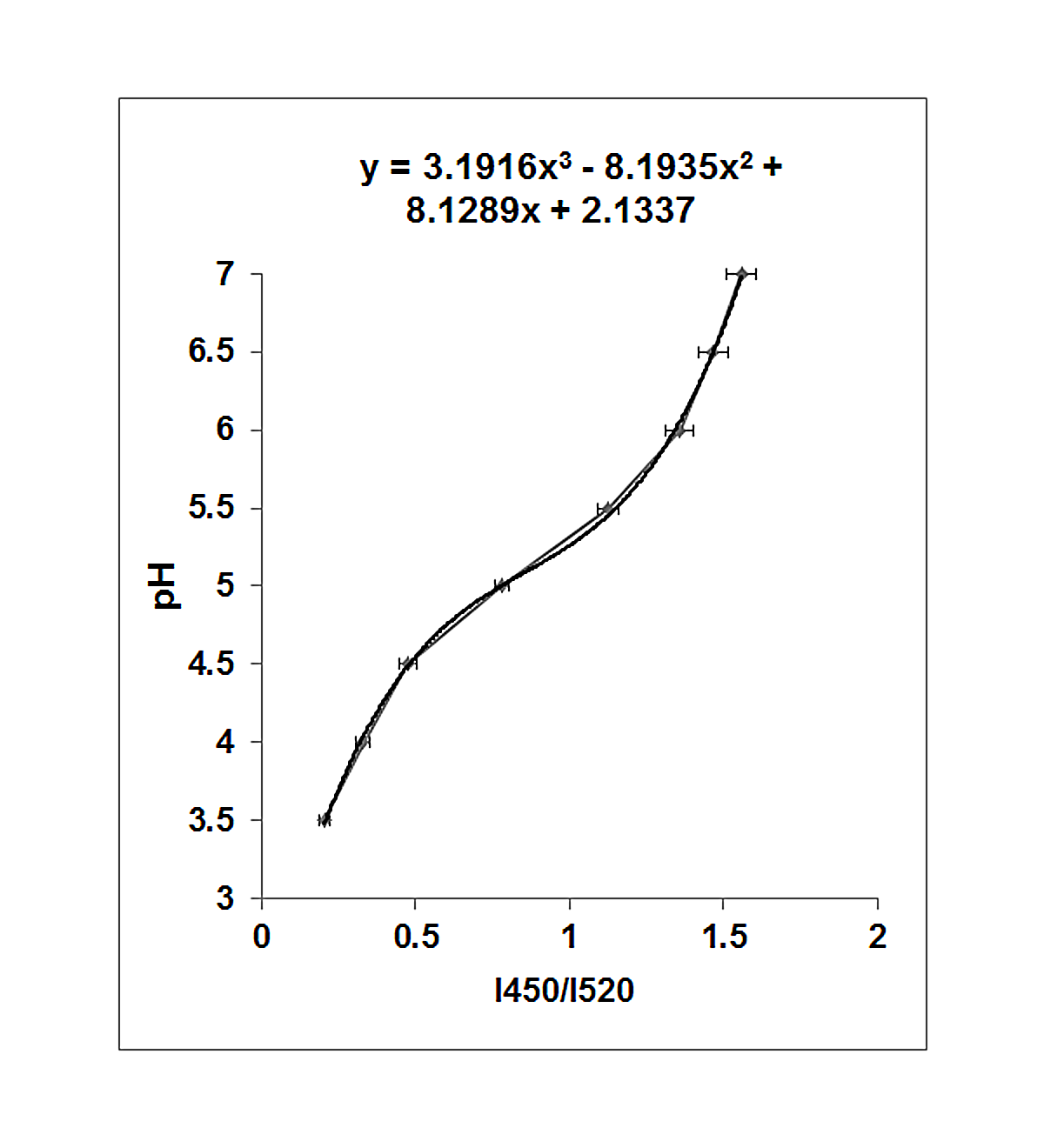

Supplement: Figure S5 — Titration curve used to convert Lysosensor yellow/blue emission (fluorescence intensity) ratios to pH values. (TIF) [file ppat.1002623.s005.tif]
