# Supplementary material for: The Exopolysaccharide Matrix Modulates the Interaction between 3D Architecture and Virulence of a Mixed-Species Oral Biofilm
Source: PLoS Pathog. 2012 Apr 5;8(4):e1002623. doi: 10.1371/journal.ppat.1002623 (PMC3320608; doi:10.1371/journal.ppat.1002623)
Supplement: Protocol S1 — Detailed methodology for 3D in situ pH mapping. (DOC) [file ppat.1002623.s006.doc]

**3D *in situ* pH mapping.** We used a fluorescent pH indicator Lysosensor yellow/blue (Molecular Probes) to determine the *in situ* pH throughout the 3D architecture. Lysosensor yellow/blue is conjugated with dextran (10kDa MW; Molecular Probes), which allows the labeled-dextran to be directly incorporated into the EPS-matrix as follows:

1. 1 μM of Lysosensor yellow/blue dextran conjugate (10kDa MW) was added to the culture medium from the beginning of the experiment and during the development of the mixed-species biofilms in 1% sucrose (with parental UA159 strain or mutant strains defective in *gtfB* and/or *gtfC*); the fluorophore does not stain the bacterial cells at the concentration used in this study, as determined experimentally.
2. Biofilms (67 h-old) were then transferred to 0.2 M/L Na_2_HPO_4_-citric acid buffer (pH 4.5; similar pH of the culture medium surrounding the biofilm at the time point of retrieval) containing 10 μM SYTO 60 (652/678; Molecular Probes) to fast (5 min) label bacterial cells. SYTO 60 was used instead of SYTO 9 to avoid fluorescence emission crosstalk with Lysosensor.
3. The first stack of images was acquired using a two photon laser scanning microscope (Olympus FV1000). For acquisition of bacterial cells images, the excitation wavelength was 810 nm, and the emission wavelength filter for SYTO 60 was HQ655/40M-2P filter. For acquisition of images from dual excitation of Lysosensor yellow/blue (incorporated into the matrix of intact biofilms), the biofilms were excited at 740 nm, and emission was detected in two channels: one at 450 nm (using 447/60 NADH bound filter) and the other at 521 nm (using 495/540 OlyMPFC1 filter).
4. After the first stack of images was acquired, the buffer was carefully removed, and 4 ml of 0.2 M/L Na_2_HPO_4-_citric acid buffer at pH 7.0 was added into the Petri dish.
5. The same stack of confocal images was acquired after 10, 30, 60 and 120 min.
6. The pH values can be measured based on fluorescence intensity ratios of the dual-wavelength fluorophore within each of the biofilm confocal image using titration curves of ratios versus pH (ranging from 3.5 to 7.0). See titration curve (Figure S5).

The preparation of titration curve of ratios versus pH included the following steps:

1. A series of tubes containing 4 ml (final volume) of 0.2 M/L Na_2_HPO_4-_citric acid buffer with pH ranging from 3.5 to 7.0 (with increments of 0.5) were prepared. A wide concentration range from 0.01 μM to 10 μM of pH indicator Lysosensor yellow/blue dextran conjugate (10,000 MW) was added to each tube containing Na_2_HPO_4-_citric acid buffer to determine if the concentration of the fluorophore affects the titration curve.

2. The 4 mL samples were transferred to Petri dishes (diameter 3.5cm) (one dish per sample). Images of standard curve solutions were acquired using a two photon laser scanning confocal microscopy Olympus FV 1000. The excitation wavelength was 740 nm, and emission was detected in two channels: 450 nm using 447/60 NADH bound filter and 521 nm using 495/540 OlyMPFC1 filter.

3. Fluorescence intensity of specific spots was analyzed using Image J (for detail see manual at http://www.uhnresearch.ca/facilities/wcif/PDF/ImageJ_Manual.pdf) and the fluorescent intensity ratio of 450 nm to 521 nm was calculated. Standard curves of pH vs. fluorescence ratio between 450nm and 521nm were plotted, and a polynomial equation was generated as reference for pH measurement in biofilms (Figure S5). The concentration of the fluorophore did not affect the titration curve, and the concentration of the fluorophore used to plot the curve in Figure S5 was 1 µM. The visualization of pH distribution within 3D biofilm architecture was performed as follows:

1. Biofilm images stack labeled with Lysosensor yellow/blue were obtained as described previously.
2. Image stacks of both channels (450nm and 521nm) were imported into Image J, using the “process” function. Image calculator was used to divide the images from channel 450nm by images from channel 521nm, and a new set of images was generated by this function. The new image stack was saved as a pH profile.
3. pH profile image stacks were loaded into Amira. Image pixel size (X, Y, and Z) was also input, which match the size of the original image stack obtained directly from confocal imaging.
4. 3D images were generated using the “voltex rendering” function in Amira. “Pure white” was chosen from the colormap in Amira to represent the color of the rendering images. Here, we chose the pure white in order to better visualize the intensity distribution of images and avoid the interference of other colors in the image intensity.

These approaches could be adapted to other biofilm systems to locate for example production of oxygen and other ions, and metabolic products.

**REFERENCES**

1. Klein MI, Duarte S, Xiao J, Mitra S, Foster TH, et al. (2009) Structural and molecular basis of the role of starch and sucrose in Streptococcus mutans biofilm development. Appl Environ Microbiol 75: 837-841.

2. DePedro HM, Urayama P (2009) Using LysoSensor Yellow/Blue DND-160 to sense acidic pH under high hydrostatic pressures. Anal Biochem 384: 359-361.
