# Supplementary material for: The Exopolysaccharide Matrix Modulates the Interaction between 3D Architecture and Virulence of a Mixed-Species Oral Biofilm
Source: PLoS Pathog. 2012 Apr 5;8(4):e1002623. doi: 10.1371/journal.ppat.1002623 (PMC3320608; doi:10.1371/journal.ppat.1002623)
Supplement: Table S1 — pH of the culture medium during mixed-species biofilm formation. (DOC) [file ppat.1002623.s007.doc]

**Time (h)**

**0.1% Sucrose**

**1% Sucrose***

**1% Glucose***

**pH**

SD

**pH**

SD

**pH**

SD

0

7.10

7.10

7.10

19

5.94

0.07

5.97

0.11

5.96

0.09

29

6.16

0.05

6.17

0.05

6.17

0.05

43

6.18

0.05

4.55

0.02

4.54

0.06

53

6.04

0.06

4.60

0.06

4.71

0.17

67

6.23

0.06

4.50

0.07

4.47

0.05

77

6.18

0.05

4.52

0.04

4.58

0.03

91

6.18

0.05

4.36

0.10

4.45

0.14

101

6.17

0.08

4.50

0.05

4.59

0.23

115

6.10

0.07

4.22

0.06

4.44

0.16

**Table S1**

**–**

**Culture pH during mixed**

**-**

**species biofilm formation**

*** Introduction of**

**1% sucrose or 1% glucose**

The data are mean values

±

standard deviations from

three

independent experiments for each condition (

*n*

=12). The pH values

(after 29h) between 1% glucose and 1% sucrose groups are not

significantly different from each other (

*P*

>0.05), but are significantly

different from 0.1% sucrose group (

*P*

<0.05, ANOVA comparison for

all pairs using

Tukey

-

Kramer HSD).

**Time (h)**

**0.1% Sucrose**

**1% Sucrose***

**1% Glucose***

**pH**

SD

**pH**

SD

**pH**

SD

0

7.10

7.10

7.10

19

5.94

0.07

5.97

0.11

5.96

0.09

29

6.16

0.05

6.17

0.05

6.17

0.05

43

6.18

0.05

4.55

0.02

4.54

0.06

53

6.04

0.06

4.60

0.06

4.71

0.17

67

6.23

0.06

4.50

0.07

4.47

0.05

77

6.18

0.05

4.52

0.04

4.58

0.03

91

6.18

0.05

4.36

0.10

4.45

0.14

101

6.17

0.08

4.50

0.05

4.59

0.23

115

6.10

0.07

4.22

0.06

4.44

0.16

**Table S**

**–**

**Culture pH during mixed**

**-**

**species biofilm formation**

*** Introduction of**

**1% sucrose or 1% glucose**

*** Introduction of**

**1% sucrose or 1% glucose**

The data are mean values

±

standard deviations from

three

independent experiments for each condition (

*n*

=12). The pH values

(after 29h) between 1% glucose and 1% sucrose groups are not

significantly different from each other (

*P*

>0.05), but are significantly

different from 0.1% sucrose group (

*P*

<0.05, ANOVA comparison for

all pairs using

Tukey

-

Kramer HSD).
