# Supplementary material for: The Exopolysaccharide Matrix Modulates the Interaction between 3D Architecture and Virulence of a Mixed-Species Oral Biofilm
Source: PLoS Pathog. 2012 Apr 5;8(4):e1002623. doi: 10.1371/journal.ppat.1002623 (PMC3320608; doi:10.1371/journal.ppat.1002623)
Supplement: Table S2 — Primers and TaqMan probes used for multiplex RT-qPCR assays. (DOC) [file ppat.1002623.s008.doc]

**Table S2**. **Primers and TaqMan probes used for multiplex RT-qPCR.**

| **GenBank Locus Tag** | **Gene Name** | **Primer sequence (forward and reverse)** | **TaqMan probe sequence and dual-labeled probes (reporters and quenchers)** |
| --- | --- | --- | --- |
|  | *16S rRNA* | ACCAGAAAGGGACGGCTAAC | CTAACGCAATAAGCACTCCGCCTGG |
|  |  | TAGCCTTTTACTCCAGACTTTCCTG | 5’ FAM / 3’ BHQ-1 |
| SMU.1004 | *gtfB* | AAACAACCGAAGCTGATAC | ATTGGCTGCATTGCTATCATCA |
|  |  | CAATTTCTTTTACATTGGGAAG | 5’ HEX / 3’ BHQ-1 |
| SMU.1005 | *gtfC* | CTCTGACTGCTACTGATACAAG | AGCAACATCTCAACCAACCGCC |
|  |  | CCGAAGTTGTTGTTGGTTTAAC | 5’ Cal Fluor Red 610 / 3’ BHQ-2 |
| SMU.910 | *gtfD* | AGCACAAACTTCTGAAGAGC | CCTGTGCTTCTTCTGCTTGCTT |
|  |  | CAGCTTTTGCCTGTGTTAAAG | 5’ Quasar 670 / 3’ BHQ-2 |
| SMU.2042c | *dexA* | TATTTTAGAGCAGGGCAATCG | ACGCCAGTCATCCTCAACCGCA |
|  |  | AACCTCCAATAGCAGCATAAC | 5’ Quasar 705 / 3’ BHQ-2 |
| SMU.78 | *fruA* | AACAACTGCTGCTGATACTG | TCTGGCTGCTGTCTTCTGTTCT |
|  |  | CTGCGGTTTCTTGAGATGAC | 5’ FAM / 3’ BHQ-1 |
| SMU.2028c | *ftf* | CTGACATAACTACGCCAAAG | CGCAATCTTACGAGCCTGTTCTGTT |
|  |  | TGCTTAAATTAATACCAGCTTC | 5’ HEX / 3’ BHQ-1 |

The primers and TaqMan probes for multiplex RT-qPCR were designed using Beacon Designer 2.0 software (Premier Biosoft International, Palo Alto, CA) (see Table S1). First, to assure an optimal multiplex RT-qPCR assay, the individual primers were optimized with SYBR green RT-qPCR assays (including determination of optimal primer concentration and melt curve analyzes). The ideal multiplex RT-qPCR should detect the internal control and the target genes at the same time. However, 16S rRNA was detected using a cDNA dilution of 1:10,000 for biofilms, and the target genes were detected using a cDNA dilution of 1:5. Therefore, 16S rRNA primers/TaqMan probe were run separately, but primers/TaqMan probes for other specific targets were combined and used in a multiplex setting. The assays were performed using a Bio-Rad CFX96 system (Bio-Rad Laboratories, Inc., CA, USA), and for reactions with only one TaqMan probe (used for target 16S rRNA) we used iQ Supermix (BioRad). For multiplex reactions (used for targets *gtfB, gtfC, gtfD* and *fruA* mixed and, for *dexA* and *ftf* mixed) we used iQ Multiplex Powermix (BioRad). The primer and probes concentrations were 250 nM and 125 nM, respectively.
